# Incidence and Risk of Thromboembolic and Cardiovascular Adverse Events with PARP Inhibitor Treatment in Patients with Metastatic Castration-resistant Prostate Cancer: A Systematic Review and Safety Meta-analysis

**DOI:** 10.1016/j.euros.2024.12.008

**Published:** 2025-01-10

**Authors:** Brigida Anna Maiorano, Martina Catalano, Chiara Mercinelli, Antonio Cigliola, Valentina Tateo, Neeraj Agarwal, Shilpa Gupta, Giandomenico Roviello, Andrea Necchi

**Affiliations:** a Department of Medical Oncology, IRCCS San Raffaele Hospital, Milan, Italy; b Department of Health Sciences, University of Florence, Florence, Italy; c Huntsman Cancer Institute, University of Utah, Salt Lake City, UT, USA; d Department of Hematology and Oncology, Taussig Cancer Institute, Cleveland Clinic Foundation, Cleveland, OH, USA

**Keywords:** PARP inhibitor, Olaparib, Niraparib, Rucaparib, Talazoparib, Metastatic castration-resistant prostate cancer, Cardiovascular toxicity, Hypertension, Thromboembolic event, Pulmonary embolism

## Abstract

**Background and objective:**

PARP inhibitor (PARPi) treatment is an effective option for patients with metastatic castration-resistant prostate cancer (mCRPC). There are few data on the cardiovascular and thromboembolic safety of these agents in mCRPC, as cardiovascular and thromboembolic adverse events (AEs) are uncommon. Our aim was to analyze the incidence and risk of major adverse cardiovascular events (MACEs), thromboembolic events, and hypertension with PARPi therapy in mCRPC.

**Methods:**

We conducted a systematic review and meta-analysis in accordance with the Preferred Reporting Items for Systematic Reviews and Meta-Analyses (PRISMA) statement. We systematically searched the PubMed, EMBASE, and Cochrane databases and the American Society of Clinical Oncology and European Society of Medical Oncology meeting abstracts for clinical trials on PARPi use in mCRPC up to March 31, 2024. We analyzed the pooled incidence of all-grade and high-grade MACEs, thromboembolic events, and hypertension, and calculated risk ratios (RRs) for PARPi versus non-PARPi treatment.

**Key findings and limitations:**

We included 11 phase 2 or 3 trials in our meta-analysis. Hypertension was the most common AE for both any-grade (17.2%) and high-grade (9.3%) events. In comparison to other treatments, PARPi was associated with significantly higher risk of high-grade MACEs (RR 2.03; *p* = 0.03) and thromboembolic events (RR 2.15; *p* = 0.002), especially venous thromboembolism (VTE; RR 2.13; *p* = 0.004) and pulmonary embolism (RR 3.60; *p* = 0.001). The risk of hypertension, any-grade MACEs, and thromboembolic AEs was not significantly higher, apart from VTE (RR 2.17; *p* = 0.01).

**Conclusions and clinical implications:**

There is higher risk of high-grade cardiovascular and thromboembolic toxicity with PARPi use in comparison to other treatments in mCRPC, although these toxicities are rare. Clinicians should be aware of this risk, especially in a population that often has comorbidities and concomitant treatments, for correct monitoring and management of these AEs.

**Patient summary:**

Drugs called PARP inhibitors are very effective in the treatment of metastatic prostate cancer that does not respond to hormone treatment. However, their use is associated with some cardiovascular adverse events, although these are rare. Our study shows that these events seem to be more frequent with PARP inhibitors than with other treatments, especially for severe grades. Doctors and patients should be aware of this risk to help in preventing, recognizing, and managing the occurrence of these rare complications.

## Take Home Message

PARP inhibitors are a very effective treatment option for metastatic castration-resistant prostate cancer. Although rare cardiovascular and thromboembolic toxicity is rare, there is a risk of high-grade adverse events with PARP inhibitors in comparison to other treatments in this setting.

## 1 Introduction

PARP inhibitor (PARPi) therapy represents an excellent example of precision medicine in prostate cancer. Since 2020, regulatory agencies have approved several agents, the first of which was for patients with metastatic castration-resistant prostate cancer (mCRPC) [1]. Olaparib was approved for patients with mCRPC carrying mutations in homologous recombination repair (HRR) genes after progression on an androgen receptor pathway inhibitor (ARPI) on the basis of results from the PROfound study [2]. Similarly, rucaparib was authorized for mCRPC with *BRCA1/2* mutations after progression on ARPI and taxane therapy on the basis of results from the TRITON2 study [3]. More recently, PARPi + ARPI combinations have been approved, such as olaparib + abiraterone in treatment-naïve mCRPC (PROpel trial) and enzalutamide + talazoparib (TALAPRO-2) [4], [5]. The rationale for PARPi therapy is synthetic lethality acquired after simultaneous occurrence of events that are not themselves lethal individually: contemporaneous defects in the HR system and loss of the counterbalancing base excision repair system lead to accumulation of double-strand breaks and eventual cell death [6].

The most frequent adverse events (AEs) associated with PARPi use in clinical trials are hematological AEs, gastrointestinal AEs, and fatigue. Hypertension and cardiovascular and thromboembolic AEs have also been reported, but were much less frequent; however, these can be of particular concern in patients with mCRPC, who are often of advanced age, have cardiovascular comorbidities, or are being treated with hormonal agents or an ARPI, which carry an additional cardiovascular risk. We conducted a systematic review and safety meta-analysis to evaluate the incidence and relative risk (RR) of hypertension and cardiovascular and thromboembolic AEs in patients with mCRPC receiving PARPi treatment.

## 2 Methods

### 2.1 Search strategy and study selection

This systematic review and meta-analysis were carried out in accordance with the Preferred Reporting Items for Systematic reviews and Meta-Analyses (PRISMA) guidelines [7]. The protocol was registered on PROSPERO (Supplementary Fig. 1).

Two authors (B.A.M. and M.C.) systematically reviewed Medline/PubMed, EMBASE, and the Cochrane Library databases, as well as American Society of Clinical Oncology and European Society of Medical Oncology meeting abstracts up to March 31, 2024. Phase 2 or 3 clinical trials that enrolled patients with mCRPC treated with PARPi for whom data on cardiovascular/thromboembolic AEs or hypertension published in English were included. Reviews, meta-analyses, commentaries, letters, personal opinions, studies not including human subjects, and studies lacking relevant safety data were excluded. Full texts and conference abstracts were examined, and reference citations were manually screened for additional candidate studies. The terms used for the database searches were PARPi, “PARP inhibitor*”, olaparib, niraparib, rucaparib, talazoparib, cardiotoxicity, “cardiovascular toxicity”, MACE, “myocardial infarction”, arrhythmia, hypertension, thromboembol*, thrombosis, “ischemic attack”, stroke, cerebrovascular, VTE, “pulmonary embolism”, “prostate cancer”, “prostate carcinoma”, and prostate. If more than one publication was found for the same trial, the most recent version was included in the final analysis (Supplementary Table 1).

The quality of the studies included in the review was assessed using the Jadad 5-item scale, taking into account randomization, methods of randomization, blinding, method of blinding, and withdrawals, with a final score ranging from 0 to 5 [8].

### 2.2 Outcomes

Our aim was to assess the incidence and RR of major adverse cardiac events (MACEs), hypertension, and thromboembolic events in patients with mCRPC treated with PARPi. Given the rarity of the different types of cardiac events expected, we decided to merge these into an overall MACE group, which included acute myocardial infarction, cardiac arrest, cardiac failure, and arrhythmias. Thromboembolic AEs included arterial and venous thromboembolism (VTE), venous thrombosis, thrombophlebitis, pulmonary embolism (PE), ischemic or hemorrhagic stroke, transient ischemic attack (TIA), and cerebrovascular accidents. For these risks, we analyzed both any-grade (grades 1–5) and high-grade (≥G3), AEs and conducted a meta-analysis to identify a significant differences between treatments (PARPi vs non-PARPi therapy). We also explored RRs for individual AEs when available in the studies.

### 2.3 Data extraction

Two authors (B.A.M. and M.C.) conducted data extraction independently, and discrepancies were resolved via consensus. The following data were extracted for each trial: name of the study and first author; year of publication; trial phase; number of patients for the safety analysis; type of treatment in each arm (experimental and control); and the number of any-grade and high-grade MACE, hypertension, and thromboembolic events in the study arms.

### 2.4 Statistical methodology

Summary incidence rates and RRs for MACE, hypertension, and thromboembolic events with 95% confidence intervals (CIs), were calculated for each study. The summary estimates were generated using the generic inverse variance approach and a fixed-effects (Mantel-Haenszel method) or random-effects (DerSimonian-Laird method) model, depending on the heterogeneity [9], [10]. The presence of heterogeneity among the studies was assessed via χ^2^ tests and the I^2^ statistic, with I^2^ values of 25%, 50%, and 75% indicating low, moderate, and high heterogeneity, respectively [10]. If I^2^ was <50%, the fixed-effects model was used; otherwise, the random-effects model was used. Subgroup analyses were planned to detect underlying sources of heterogeneity in terms of the PARPi administered (olaparib, niraparib, rucaparib, talazoparib), treatment regimen (PARPi monotherapy vs combination), and disease setting (treatment-naïve vs pretreated patients). A sensitivity analysis was performed to assess the stability of the global estimate by excluding one study at a time. Publication bias was not evaluated because the total number of studies included was less than ten. Statistical significance was set at *p* < 0.05 and all tests were two-sided. No correction for multiplicity was applied. R Studio was used to perform the statistical analysis.

## 3 Results

### 3.1 Study selection process

The search of databases and conference abstracts identified 872 studies. After removal of duplicates, 841 papers were screened. Of these, 830 were excluded (not in English, preclinical articles or animal studies, reviews, commentaries, personal opinions, and case reports). Thus, 11 studies were included in the qualitative analysis. Among these, the meta-analysis was restricted to the six randomized controlled trials (RCTs; Supplementary Fig. 1).

### 3.2 Characteristics of the studies

The qualitative analysis included 11 studies [2], [3], [11], [12], [13], [14], [15], [16], [17], [18], [19], [20]. Of these, four were single-arm open-label phase 2 studies investigating olaparib (*n* = 1), niraparib (*n* = 1), talazoparib (*n* = 1), and rucaparib (*n* = 1) [3], [17], [18], [19]. One phase 2 RCT was only included in the qualitative synthesis, as patients in the experimental arm and the control arm received olaparib, given at different dosages (300 vs 400 mg) [16]. The other six RCTs were included in the quantitative meta-analysis. Of these, two were phase 2 and four were phase 3 trials [2], [20], [11], [12], [13], [14], [15]. In two studies, PARPi monotherapy in the experimental arm (olaparib and rucaparib) was compared to an ARPI and to ARPI or docetaxel, respectively, in the control arm [2], [11]. In four studies, combined PARPi + ARPI in the experimental arm was compared to ARPI + placebo in the control arm. Combinations included olaparib + abiraterone (*n* = 2), niraparib + abiraterone (*n* = 1), and talazoparib + enzalutamide (*n* = 1) [20], [12], [13], [14], [15]. Overall, the quality of the studies was good (Table 1 and Supplementary Table 1).

**Table 1:** Characteristics of the studies included in the review

Study and year	Phase	Disease setting	Treatment arms	Patients per arm	RoB
PROFound (NCT02987543) 2020 [2]	3	mCRPC (post-ARPI)	Olaparib 300 mg BID	162	3
			ARPI	83	
TRITON3 (NCT02975934) 2023 [11]	3	mCRPC (post-ARPI)	Rucaparib 600 mg BID	270	3
			ARPI	135	
			CTx	75	
Study 08 (NCT01972217) 2018 [12]	2	mCRPC (post-Txt)	Olaparib 300 mg + Abi	71	5
			Abiraterone + PBO	71	
MAGNITUDE (NCT03748641) 2022 [13]	3	mCRPC (treatment-naïve)	Niraparib 200 mg OD + Abi	212	5
			Abiraterone + PBO	211	
PROpel (NCT03732820) 2022 [20]	3	mCRPC (treatment-naïve)	Olaparib 300 mg BID + Abi	399	5
			Abi + PBO	397	
			Olaparib 300 mg BID + Abi	21	
TALAPRO-2 (NCT03395197) 2023 [14], [15]	3	mCRPC (treatment-naïve)	Talazoparib 0.5 mg OD + Enza	402	5
			PBO + Enza	402	
TOPARP-B (NCT01682772) 2020 [16]	2	mCRPC (post-taxane)	Olaparib 300 mg BID	49	3
			Olaparib 400 mg BID	49	
TOPARP-A (NCT01682772) 2015 [17]	2	mCRPC (post-CTx)	Olaparib 400 mg BID	50	1
TRITON2 (NCT02952534) 2020 [3]	2	mCRPC (post-ARPI and Txt)	Rucaparib 600 mg BID	115	1
TALAPRO-1 (NCT03148795) 2021 [18]	2	mCRPC (post-ARPI and Txt)	Talazoparib 1 mg OD	127	1
GALAHAD (NCT02854436) 2022 [19]	2	mCRPC (post-ARPI and Txt)	Niraparib 300 mg OD	223 a	1

Abi = abiraterone; ARPI = androgen receptor pathway inhibitor; BID = twice daily; CTx = chemotherapy; mCRPC = metastatic castration-resistant prostate cancer; Enza = enzalutamide; NA = not available; OD = once daily; PBO = placebo; RoB = risk of bias; Txt = Taxotere.

a 142 patients with a *BRCA* mutation.

### 3.3 AE incidence rates

Data from 11 studies (2195 patients) were available for calculating the overall incidence of the different AEs. Among the AEs, hypertension was the most frequent (17.2%), and was of grade ≥3 in 9.3% of patients. MACEs and thromboembolic events were less frequent in terms of both of any-grade (5.2% and 3.8%) and high-grade (2.1% and 3.3%) AEs (Table 2).

**Table 2:** Pooled incidence of hematologic adverse events with PARPi versus non PARPi therapy in metastatic castration-resistant prostate cancer

Adverse events by grade	Incidence rate, % (range)	
	PARPi	Non-PARPi
**Any-grade adverse events**		
Hypertension	17.2 (5.9–50.9)	14.1 (5,2–34.1)
Major adverse cardiac events	5.2 (0.8–16.5)	5.0 (2.3–11.4)
Thromboembolic adverse events	3.8 (3.1–5.5)	3.5 (0–7.7)
**Grade ≥3 adverse events**		
Hypertension	9.3 (1.17–25.1)	6.7 (0–18.0)
Major adverse cardiac events	2.1 (0.4–8.5)	1.3 (0–4.7)
Thromboembolic adverse events	3.3 (0.3–8.0)	1.8 (0.5–4.8)

PARPi = PARP inhibitor.

### 3.4 Hypertension

Data on the RR of hypertension of any grade were available from five studies: in total, 1349 patients received PARPi therapy in the experimental arm and 1209 received other therapy in the control arm. Data on grade ≥3 hypertension were available from six studies for 2914 patients (1605 in the PARPi group).

**Fig. 1:**
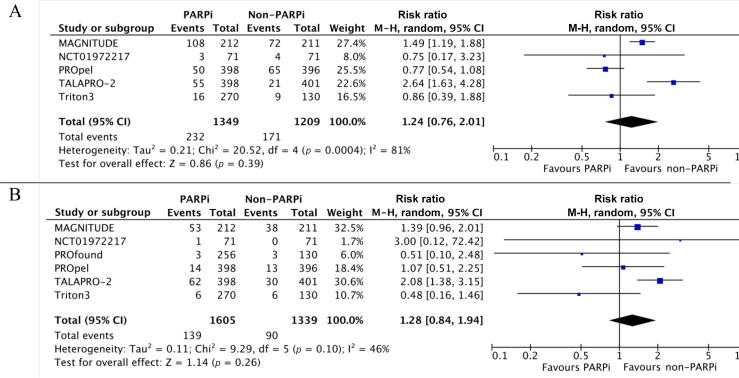
Relative risk of hypertension with PARP inhibitor (PARPi) versus non-PARPi therapy in metastatic castration-resistant prostate cancer: (A) any-grade and (B) high-grade hypertension. CI = confidence interval; df = degrees of freedom; M-H = Mantel-Haenszel method.

PARPi use did not significantly increase the risk of any-grade (RR 1.24, 95% CI 0.76–2.01; *p* = 0.39) or grade ≥3 hypertension (RR 1.28, 95% CI 0.84–1.94; *p* = 0.26). There was high heterogeneity among the studies for the any-grade (I^2^ = 81%) and grade ≥3 (I^2^ = 46%) analyses (Fig. 1A, B).

### 3.5 MACEs

Three studies were included in the RR analysis for any-grade MACEs (681 patients in the PARPi arm) and five studies in the analysis for grade ≥3 MACEs (1207 patients in the PARPi arm).

**Fig. 2:**
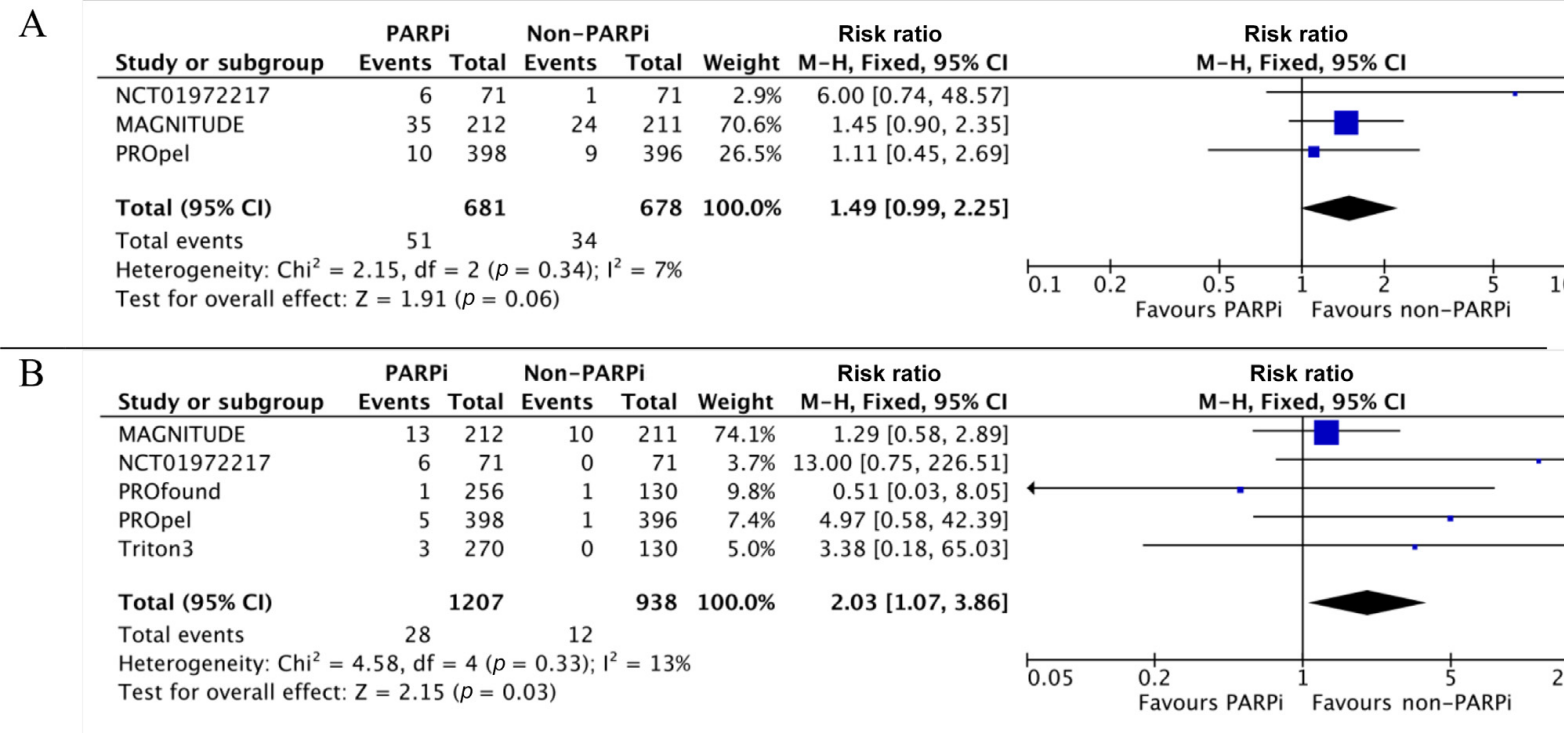
Relative risk of major adverse cardiac events with PARP inhibitor (PARPi) versus non-PARPi therapy in metastatic castrate-resistant prostate cancer: (A) any-grade and (B) high-grade events. CI = confidence interval; df = degrees of freedom; M-H = Mantel-Haenszel method.

The risk of any-grade MACEs did not significantly differ between PARPi and non-PARPi therapy (RR 1.49, 95% CI 0.99–2.25; *p* = 0.06). However, the risk of grade ≥3 MACEs was significantly higher with PARPi than with non-PARPi therapy (RR 2.03, 95% CI 1.07–3.86; *p* = 0.03). There was low heterogeneity among the studies for these analyses (Fig. 2A, B).

No significant differences between PARPi and non-PARPi therapy emerged when we analyzed individual AEs, and there was no decrease in heterogeneity (Supplementary Fig. 2).

### 3.6 Thromboembolic events

The five studies included in the analysis of any-grade thromboembolic AEs involved a total of 2802 patients (1534 in the experimental arm and 1268 in the control arm). For analysis of grade ≥3 AEs five studies involving 2544 patients (1335 in the PARPi arm) had data available.

**Fig. 3:**
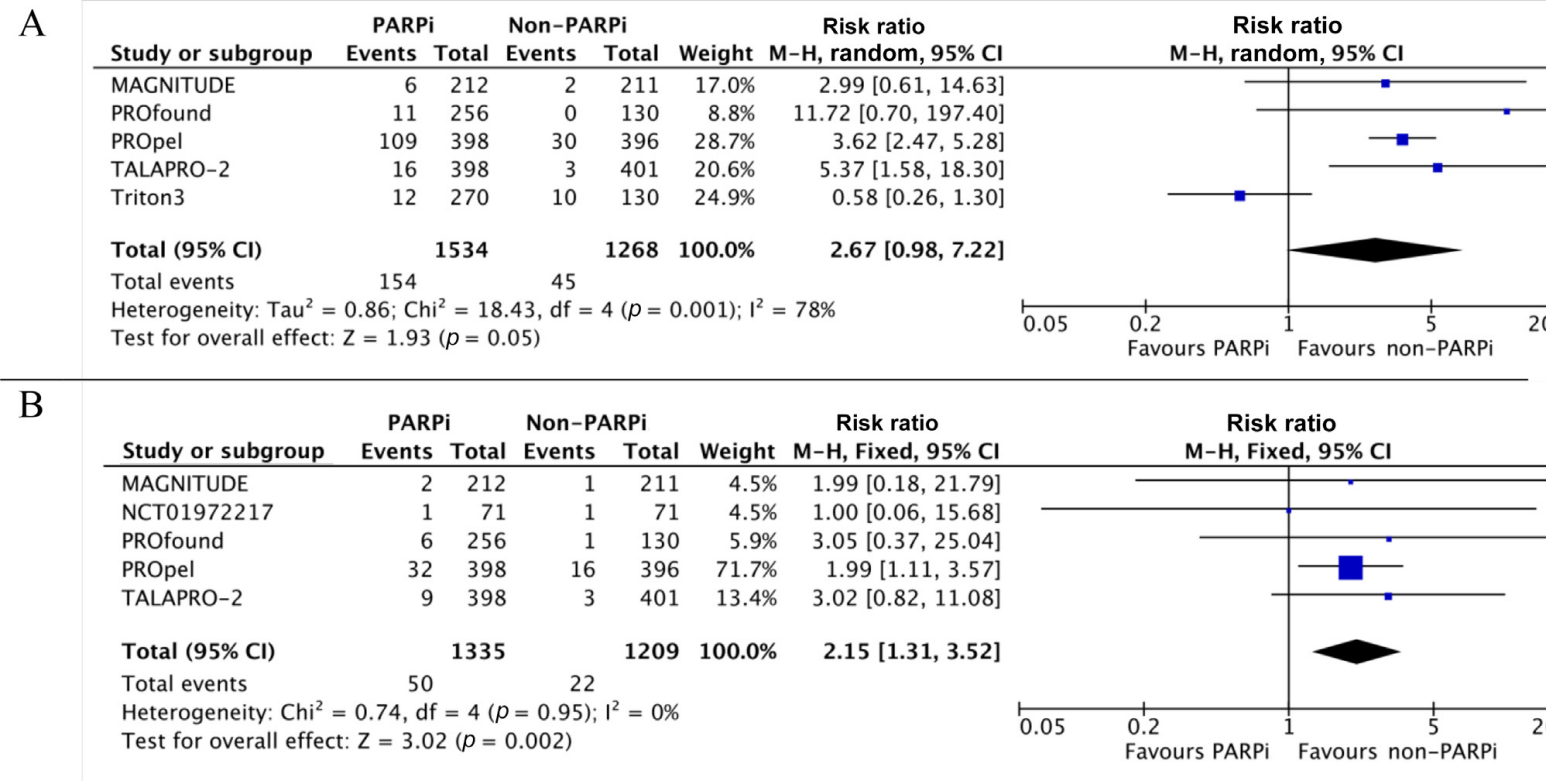
Relative risk of thromboembolic events with PARP inhibitor (PARPi) versus non-PARPi therapy in metastatic castration-resistant prostate cancer: (A) any-grade and (B) high-grade events. CI = confidence interval; df = degrees of freedom; M-H = Mantel-Haenszel method.

PARPi did not significantly increase the risk of thromboembolic events of any grade in comparison to other treatments (RR 2.67, 95% CI 0.98–7.22; *p* = 0.05). There was significantly high heterogeneity among the studies (I^2^ = 81%; *p* = 0.001). The risk of grade ≥3 thromboembolic events was significantly higher for the PARPi arm than for the control arm (RR 2.15, 95% CI 1.31–3.52; *p* = 0.002). The studies included in the grade ≥3 analysis were homogeneous (I^2^ = 0%; Fig. 3A, B).

We then analyzed the risk of developing PE (3 studies) and VTE (4 studies) separately. PARPi use significantly increased the risk of grade ≥3 PE (RR 3.60, 95% CI 1.67–7.75; *p* = 0.001; I^2^ = 0%). PARPi use also significantly increased the risk of any-grade VTE (RR 2.17, 95% CI 1.20–3.90; *p* = 0.01) and high-grade VTE (RR 2.13, 95% CI 1.27–3.58; *p* = 0.004), with low heterogeneity (I^2^ = 18% and 0%, respectively; Supplementary Fig. 2).

### 3.7 Subgroup and sensitivity analyses

To explore sources of heterogeneity, we performed subgroup analyses according to drug type (olaparib, niraparib, rucaparib, talazoparib), PARPi monotherapy versus combination with ARPI, and disease setting (treatment-naïve vs pretreated mCRPC). Different strategies reduced or eliminated the heterogeneity among the studies, including the PARPi agent used for MACEs and grade ≥3 thromboembolic AEs; and the administration setting (treatment-naïve vs pretreated mCRPC) and strategy (PARPi monotherapy vs combination) for all the AEs, apart from grade ≥3 hypertension. Some subgroup analyses revealed significant differences in AE RRs. The risk of any-grade hypertension (more significant increases with niraparib and talazoparib) and thromboembolic AEs (increased with olaparib and talazoparib) differed according to the PARPi agent used, and the risk of grade ≥3 hypertension differed with the disease setting (all *p* < 0.05). The other subgroup analyses did not impact heterogeneity (Table 3).

**Table 3:** Subgroup analyses for hypertension, MACEs, and thromboembolic adverse events with PARPi therapy in metastatic castration-resistant prostate cancer^a^

Subgroup	Relative risk (95% confidence interval)					
	Any-grade adverse events			Grade ≥3 adverse events		
	HTN	MACE	TE/PE	HTN	MACE	TE/PE
**PARPi**						
Olaparib	0.76 (0.55–1.07)	1.97 (0.40–9.73)	3.69 (2.53–5.38)	0.98 (0.51–1.90)	4.37 (1.16–16.51)	1.99 (1.15–3.46)
Niraparib	1.49 (1.19–1.88)	1.45 (0.90–2.35)	2.99 (0.61–14.63)	1.39 (0.96–2.01)	1.31 (0.56–3.06)	1.99 (0.18–21.79)
Rucaparib	0.86 (0.39–1.88)	–	0.58 (0.26–1.30)	0.48 (0.16–1.46)	3.41 (0.18–66.60)	–
Talazoparib	2.64 (1.63–2.48)	–	5.37 (1.58–18.30)	–	–	3.02 (0.82–11.08)
Subgroup differences	***p*** = **0.0001** **I^2^** = **85%**	*p* = 0.72 I^2^ = 0%	***p*** = **0.0005** **I^2^** = **83.0%**	*p* = 0.17 I^2^ = 43.3%	*p* = 0.30 I^2^ = 16.4%	*p* = 0.84 I^2^ = 0%
**Disease setting**						
Naive	1.42 (0.78–2.58)	1.36 (0.89–2.09)	3.70 (2.60–5.27)	1.55 (1.10–2.20)	1.75 (0.58–5.30)	2.13 (1.27–3.58)
Pre-treated	0.83 (0.41–1.66)	6.00 (0.74–48.57)	2.03 (0.08–53.52)	0.56 (0.23–1.35)	2.73 (0.39–18.89)	2.02 (0.38–10.75)
Subgroup differences	*p* = 0.25 I^2^ = 23.2%	*p* = 0.17 I^2^ = 46.0%	*p* = 0.72 I^2^ = 0%	***p*** = **0.03** **I^2^** = **77.7%**	*p* = 0.69 I^2^ = 0%	*p* = 0.95 I^2^ = 0%
**PARPi strategy**						
Monotherapy	0.86 (0.39–1.88)	–	2.03 (0.08–53.52)	0.49 (0.20–1.22)	1.23 (0.16–9.26)	3.05 (0.37–25.04)
Combination^b^	1.33 (0.76–2.31)	–	3.70 (2.60–5.27)	1.23 (0.76–2.00)	2.75 (0.69–10.99)	2.07 (1.24–3.45)
Subgroup differences	*p* = 0.37 I^2^ = 0%	–	*p* = 0.72 I^2^ = 0%	*p* = 0.08 I^2^ = 67.2%	*p* = 0.52 I^2^ = 0%	*p* = 0.73 I^2^ = 0%

CI = confidence interval; HTN = hypertension; MACE = major cardiac adverse event; PARPi = PARP inhibitor; RR = relative risk; TE/PE = thromboembolic adverse event/pulmonary embolism.

a Statistically significant differences are in bold font.

b PARPi combined with an androgen receptor pathway inhibitor.

The results did not change after exclusion of one study at a time in the sensitivity analysis for all the outcomes (Supplementary Fig. 3).

## 4 Discussion

### 4.1 Summary of the findings

**Fig. 4:**
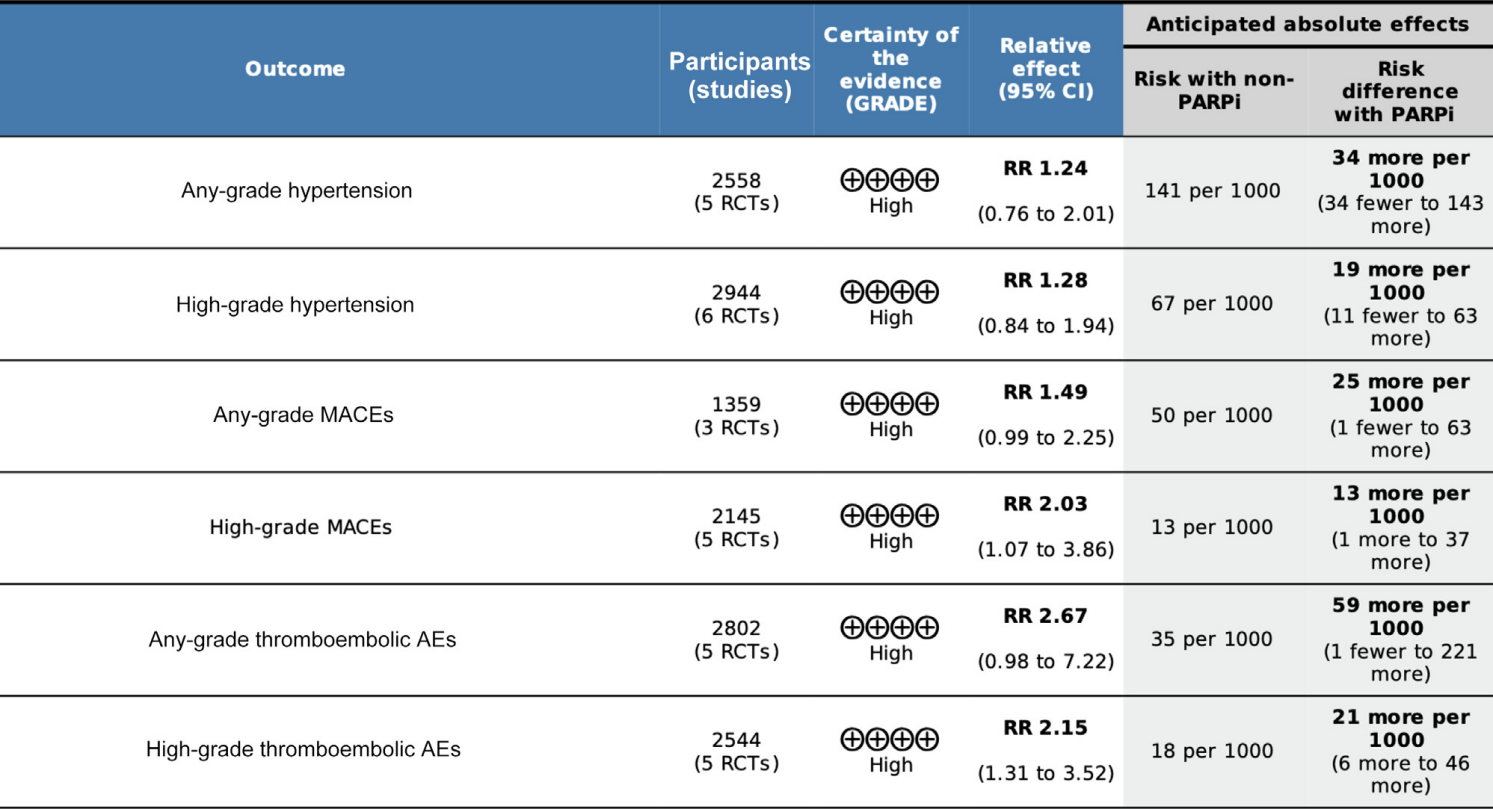
Summary of findings from the studies for hypertension, major cardiac adverse events (MACEs), and thromboembolic adverse events (AEs) with PARP inhibitor (PARPi) versus non-PARPi therapy in metastatic castration-resistant prostate cancer. CI = confidence interval; RCT = randomized clinical trial; RR = risk ratio.

To the best of our knowledge, this is the first systematic review and meta-analysis providing data on the risk of cardiovascular AEs, thromboembolic AEs, and hypertension for patients receiving PARPi therapy for mCRPC. Among patients receiving PARPi treatment, almost one in five develop any-grade hypertension, and one in ten develop high-grade hypertension. However, the RR is not significantly higher in comparison to other treatments. These findings confirm literature data for other tumor types and different PARPi agents at various dosages that indicate that these drugs are not significantly associated with a higher risk of hypertension in comparison to other treatments [21], [22]. Nevertheless, there is an increase in the risk of hypertension that ranges from 19 to 34 per 1000 patients, depending on severity. PARPi use appears to significantly increase the risk of high-grade MACEs and thromboembolic AEs, with 13 and 21 more patients, respectively, experiencing these AEs in every 1000 treated. However, these AEs are rare, with a maximum incidence of approximately 5% for any-grade AEs and 3% for high-grade AEs in our analysis. Although there is higher incidence of 25 and 59 more any-grade MACEs and thromboembolic AEs per 1000 patients treated with PARPi, the risk is not significantly higher than with non-PARPi regimens. We found that the quality of the evidence available is high, so we are confident that the true PARPi effects on AEs are close to the estimated effects (Fig. 4).

The pathogenesis of cardiovascular and thromboembolic PARPi toxicities is not completely clear. However, off-target PARPi effects on proteins other than PARP can at least partly explain the cardiovascular effects. PARPi agents, especially, niraparib, interfere with transporters of dopamine, norepinephrine, and serotonin, inhibiting their cellular uptake and disrupting the metabolism of these neurotransmitters [23]. This effect is reinforced by inhibition of DYRK1A, which contributes to the development of hypertension [24]. There are conflicting data regarding the correlation between PARPi use and arrhythmias. An analysis showed that niraparib has an off-target pro-arrhythmogenic effect as it inhibits the Kv11.1 (hERG) potassium channel, with an increase in the risk of QTc prolongation and thus arrhythmias [25]. Effectively, the structure of both niraparib and rucaparib means that they have potential pro-arrhythmogenic activity, although they are weak hERG inhibitors [26]. This is particularly true for patients with a pre-existing long QT interval [27]. However, published data regarding this higher risk differ [28]. The PARP1 pathway has been linked to myocardial alteration, hypertension, and activation of the angiotensin-II pathway, which has a detrimental effect on myocardial tissue activation as a response to ischemia/reperfusion, and on nicotinamide adenine dinucleotide (NAD^+^), which is involved in cardiomyocyte function in atrial fibrillation, with a cardioprotective effect hypothesized [29]. Similar mechanisms, such as rescue levels of NAD^+^ via the CX43-PARP1 pathway, may exert a neuroprotective effect [30], [31]. Our subgroup analyses revealed no differences in risk for the different PARPi agents used. Analysis of PARPi monotherapy versus combinations (for which ARPIs were the most common) revealed no effect of the combinations on any-grade or high-grade AEs. In addition, PARPi use in pretreated patients with potential for a cumulative effect did not seem to affect the risk of cardiovascular and thromboembolic AEs.

For other PARPi-mediated AEs, such as hematologic AEs, published data indicate that these class effects occur early after PARPi initiation [32]. Unfortunately, the studies included in our analysis did not report the time of onset or the mean duration of cardiovascular and thromboembolic AEs. Regarding the relationship between these AEs and patient outcomes, cardiorespiratory failure caused two deaths and acute cardiac failure caused another death in the PROfound trial [2]. In TRITON3, two patients died of cardiovascular AEs during rucaparib treatment (1 acute myocardial infarction and 1 cardiac failure) [11]. In PROpel, thromboembolic toxicity of the olaparib + abiraterone combination resulted in a discontinuation rate of 1.5% and one death [20]. No more data on rates of discontinuation or dosage modifications because of these AEs have been reported.

The newest generation of PARPi agents, characterized by higher PARP selectivity, may result in lower toxicity, as indicated by preclinical models [33]. Saruparib is a first-in-class PARP1 inhibitor with exquisite PARP1 selectivity that is being tested in the phase 1/2a PETRA trial in pretreated solid tumors, including prostate cancer, with a *BRCA*, *RAD51*, or *ATM* mutation. Saruparib has shown efficacy and a good safety profile according to an interim analysis presented at the 2024 American Association for Cancer Research meeting [34]. No cardiovascular and thromboembolic safety data have been released yet, but longer follow-up and further studies should provide more information.

### 4.2 Limitations and future directions

Our systematic review and meta-analysis have several limitations. First, the analyses are based on aggregate rather than individual patient data. Moreover, there is a lack of information for separate cardiovascular and thromboembolic AE classes owing to the scarcity of data and the low numbers of events. Given the rarity of these AEs, we had to group them into categories for our primary analysis. Schemes for systematic classification and recording of these AEs could help in better identification and analysis of their incidence. Moreover, no data were available regarding baseline cardiac comorbidities and previous treatments, including androgen deprivation therapy (ADT) and ARPI therapy, which might have influenced the risk of cardiovascular and thromboembolic toxicities. This is an important limitation of our meta-analysis. We found no evidence of an interaction between AEs and the treatment setting (naïve vs pretreatment). However, a common previous regimen for the pretreated patients was ADT and an ARPI, but no data could be retrieved regarding the treatment duration and the possible impact on cardiovascular toxicity. Thus, our results might only partly reflect the real incidence of these AEs, as patients in routine clinical practice are often older with more cardiovascular comorbidities and multiple concomitant treatments in comparison to patients selected in clinical trials. Real-world evidence indicates that the risk of several cardiovascular AEs increases from five- to 17-fold with PARPi therapy, with hypertension and arrhythmias the most common AEs [35]. Regulatory agencies recommend monitoring of arterial pressure and checking baseline cardiovascular risk, particularly with niraparib, and the use of standard antihypertensive treatments if hypertension develops [36]. However, no further indications for cardiovascular or thromboembolic risk evaluations are available as no causal relationship has been identified and no increase in the incidence of these AEs with PARPi therapy in comparison to the general mCRPC population has been considered.

### 4.3 Implications for clinical practice

Patients in the clinical trials included in our review were highly selected. They usually underwent complete cardiovascular and thromboembolic screening, with severe cardiovascular comorbidities a common exclusion criterion for RCTs. This population differs from the mCRPC population in routine clinical practice, which is often characterized by multiple comorbidities, concomitant treatments, and frailty. In the advanced disease setting, for which treatment options are even more limited, careful consideration of these options is required, including the incidence of less frequent AEs, such cardiovascular and thromboembolic AEs. Guidelines do not recommend any specific monitoring. However, careful evaluation of patients with risk factors such as coexisting cardiovascular comorbidities and previous therapy with an ARPI or long-lasting ADT is warranted. Baseline and periodic re-evaluation of cardiovascular and thromboembolic risk factors could be recommended for patients considering PARPi therapy in early or later lines or when combined with an ARPI, especially because of possible additive effects on the cardiovascular system. Patients should be evaluated using proper cardiovascular and thromboembolic risk assessments and referred to a cardiologist for baseline and periodic evaluation. Prevention of this risk has been recognized as a challenge by cardiology societies [37], and trials addressing this issue are ongoing. Lifestyle interventions could be part of an integrated approach to preventing or treating these AEs.

## 5 Conclusions

In conclusion, our meta-analysis confirms that PARPi treatment is associated with an increase in the risk of high-grade cardiovascular AEs and PE, and VTE of any grade, although these AEs are rare. Clinicians should be aware of this risk for proper monitoring of symptoms and diagnosis and management of these AEs if they occur. Further research with higher numbers of patients and events is warranted to better identify the risk for individual patients receiving PARPi therapy for mCRPC.

  ***Author contributions:*** Brigida Anna Maiorano had full access to all the data in the study and takes responsibility for the integrity of the data and the accuracy of the data analysis.

  *Study concept and design*: Maiorano.

*Acquisition of data*: Maiorano.

*Analysis and interpretation of data*: Maiorano.

*Drafting of the manuscript*: Maiorano, Catalano.

*Critical revision of the manuscript for important intellectual content*: Mercinelli, Cigliola, Tateo, Agarwal, Gupta, Roviello.

*Statistical analysis*: Maiorano.

*Obtaining funding*: None.

*Administrative, technical, or material support*: Maiorano.

*Supervision*: Necchi.

*Other*: None.

  ***Financial disclosures:*** Brigida Anna Maiorano certifies that all conflicts of interest, including specific financial interests and relationships and affiliations relevant to the subject matter or materials discussed in the manuscript (eg, employment/affiliation, grants or funding, consultancies, honoraria, stock ownership or options, expert testimony, royalties, or patents filed, received, or pending), are the following: None.

  ***Funding/Support and role of the sponsor:*** None.
