# Supplementary material for: Incidence and Risk of Thromboembolic and Cardiovascular Adverse Events with PARP Inhibitor Treatment in Patients with Metastatic Castration-resistant Prostate Cancer: A Systematic Review and Safety Meta-analysis
Source: Eur Urol Open Sci. 2025 Jan 10;72:1–9. doi: 10.1016/j.euros.2024.12.008 (PMC11772952; doi:10.1016/j.euros.2024.12.008)
Supplement: Supplementary Data 1 [file mmc1.docx]

| **Population** | Patients with mCRPC |
| --- | --- |
| **Intervention** | PARPis |
| **Comparator** | Non-PARPis |
| **Outcome(s)** | RR with 95% CI of major adverse cardiovascular events, thromboembolic events, hypertension |
| **Studies** | Phase II or III clinical trials |

**Supplementary Table 1. PICOS structure for study inclusion in the meta-analysis.**

CI: confidence interval; mCRPC: metastatic castration-resistant prostate cancer; PARPis: Poly(ADP-ribose) Polymerase Inhibitors; RR: risk ratio

**Supplementary Figure 1. Preferred Reporting Items of Systematic reviews and Meta-Analysis (PRISMA) flow chart of the selection process.**

| **Study (clinicaltrial reg nr), Year** | **Randomization** | **Method of randomization** | **Blinding** | **Method of blinding** | **Withdrawals** | **Overall score** |
| --- | --- | --- | --- | --- | --- | --- |
| PROFound (NCT02987543), 2020(de Bono et al., 2020) | 2 | 0 | 0 | 0 | 1 | 3 |
| TRITON3 (NCT02975934), 2023(Fizazi et al., 2023) | 2 | 0 | 0 | 0 | 1 | 3 |
| Study 08 (NCT01972217), 2018(Clarke et al., 2018) | 2 | 0 | 2 | 0 | 1 | 5 |
| MAGNITUDE (NCT03748641), 2022(Chi et al., 2022) | 2 | 0 | 2 | 0 | 1 | 5 |
| PROpel (NCT03732820), 2022(Clarke et al., 2022) | 2 | 0 | 2 | 0 | 1 | 5 |
| TALAPRO-2 (NCT03395197), 2023(Agarwal et al., 2021; Azad et al., 2023) | 2 | 0 | 2 | 0 | 1 | 5 |
| TOPARP-B (NCT01682772), 2020(Mateo et al., 2020) | 2 | 0 | 0 | 0 | 1 | 3 |
| TOPARP-A (NCT01682772), 2015(Mateo et al., 2015) | 0 | 0 | 0 | 0 | 1 | 1 |
| TRITON2 (NCT02952534), 2020(Abida et al., 2020) | 0 | 0 | 0 | 0 | 1 | 1 |
| TALAPRO-1 (NCT03148795), 2021(De Bono et al., 2020) | 0 | 0 | 0 | 0 | 1 | 1 |
| GALAHAD (NCT02854436), 2022(Smith et al., 2019) | 0 | 0 | 0 | 0 | 1 | 1 |

**Supplementary Table 2. JADAD score of the included studies.**

| **AE** | **Nr of studies** | **Incidence rate, % (range)** | **RR (95% CI)** | **p-value** | **I2, %** |
| --- | --- | --- | --- | --- | --- |
| All-grades IMA | 2 | 6.4 (1.0-16.5) | 1.44 (0.92-2.28) | 0.12 | 0 |
| ≥G3 IMA | 4 | 2.3 (0.4-5.6) | 1.38 (0.67-2.84) | 0.39 | 0 |
| All-grades cardiac failure | 2 | 2.6 (1.5-8.4) | 2.12 (0.46-9.88) | 0.34 | 44 |
| ≥G3 cardiac failure | 3 | 1.1 (0.4-2.8) | 3.75 (0.81-17.37) | 0.09 | 0 |
| All-grades VTE | 4 | 4.8 (1.1-9.3) | **2.17 (1.20-3.90)** | **0.01** | 18 |
| ≥G3 VTE | 3 | 3.2 (0.9-8.0) | **2.13 (1.27-3.58)** | **0.004** | 0 |
| All-grades PE | 3 | 2.2 (0-4.3) | 1.07 (0.12-9.95) | 0.95 | 66 |
| ≥G3 PE | 2 | 4.9 (2.3-6.5) | **3.60 (1.67-7.75)** | **0.001** | 0 |

**Supplementary Table 3. Incidence and RR of single cardiovascular/thromboembolic AEs with PARPis in mCRPC.** Statistically significant differences are bolded.

CI: confidence interval; ≥G3: equal to over grade 3; RR: relative risk; PE: pulmonary embolism; VTE: venous thromboembolism

| A | 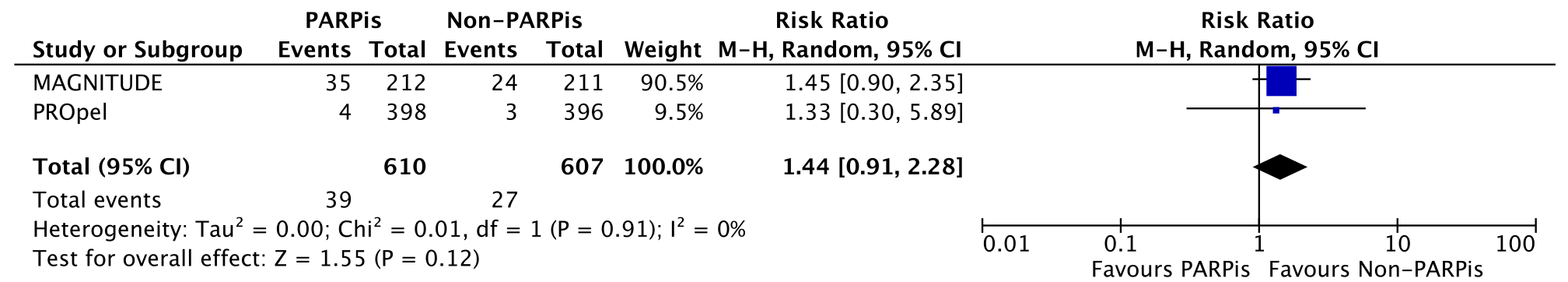 |
| --- | --- |
| B | 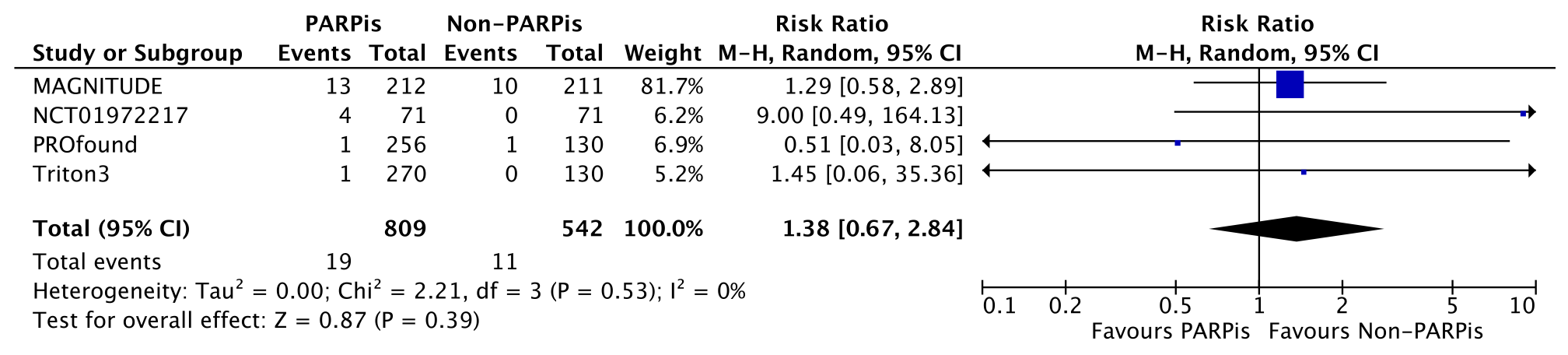 |
| C | 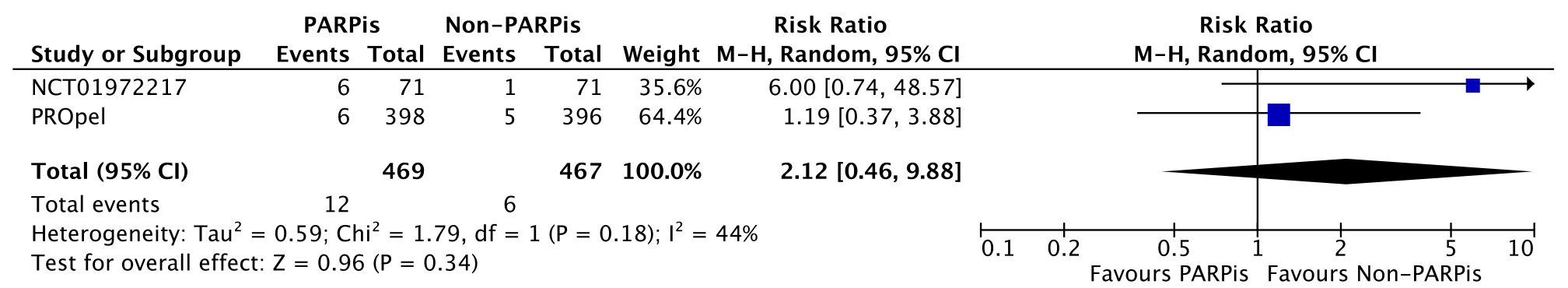 |
| D | 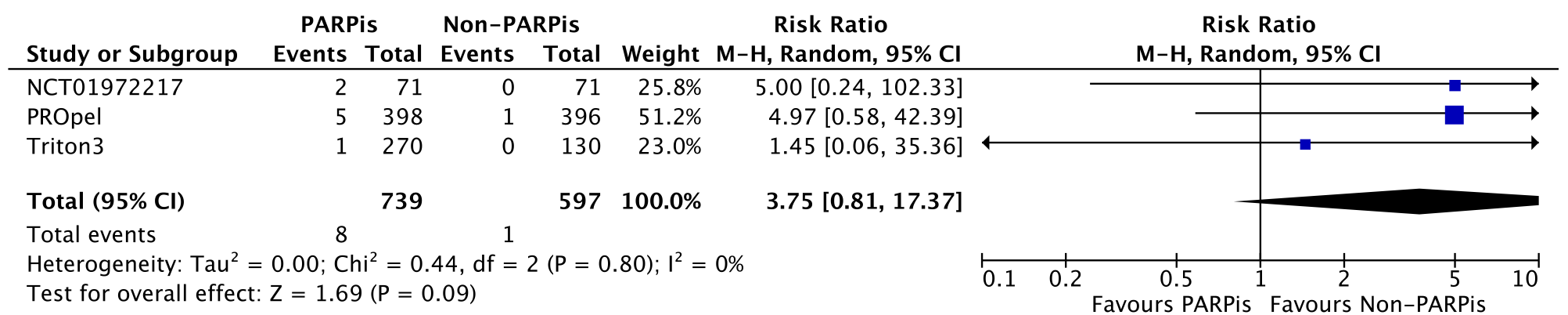 |
| E | 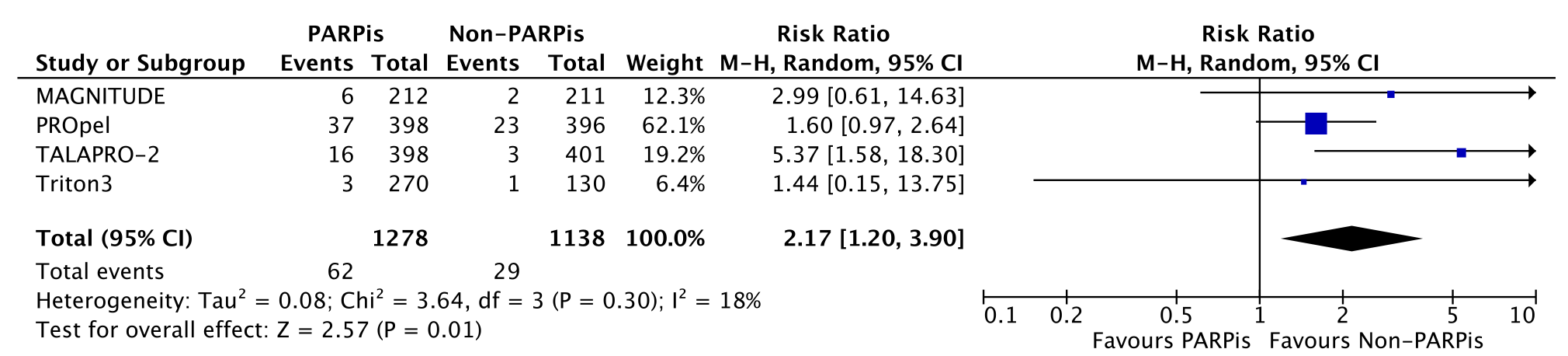 |
| F | 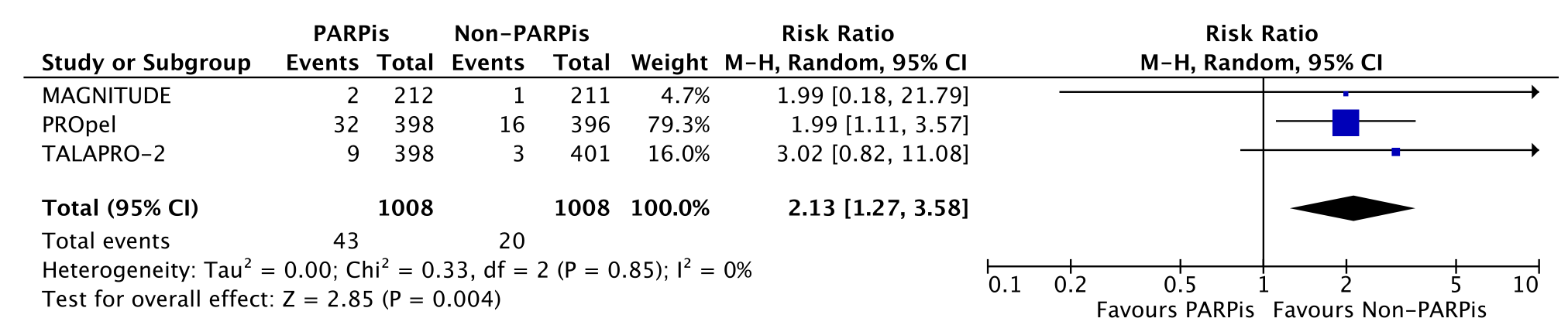 |
| G | 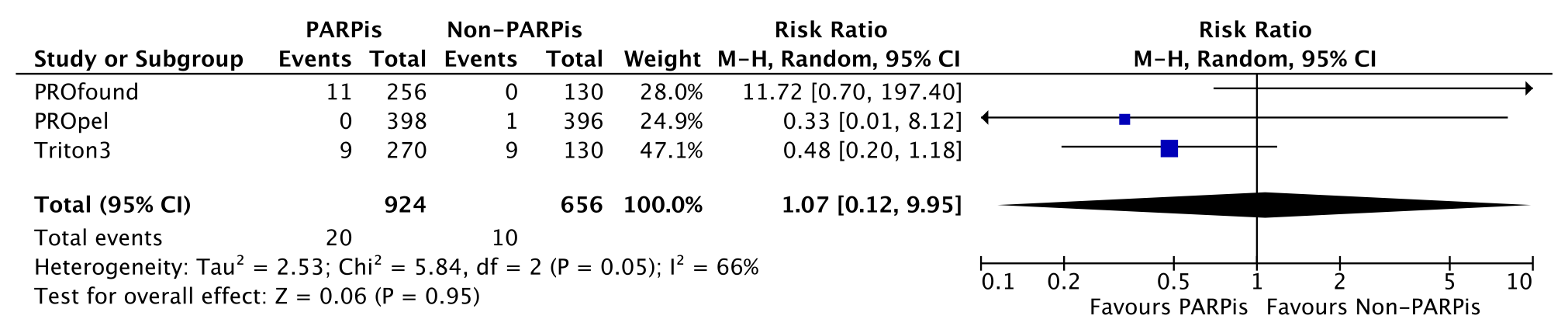 |
| H | 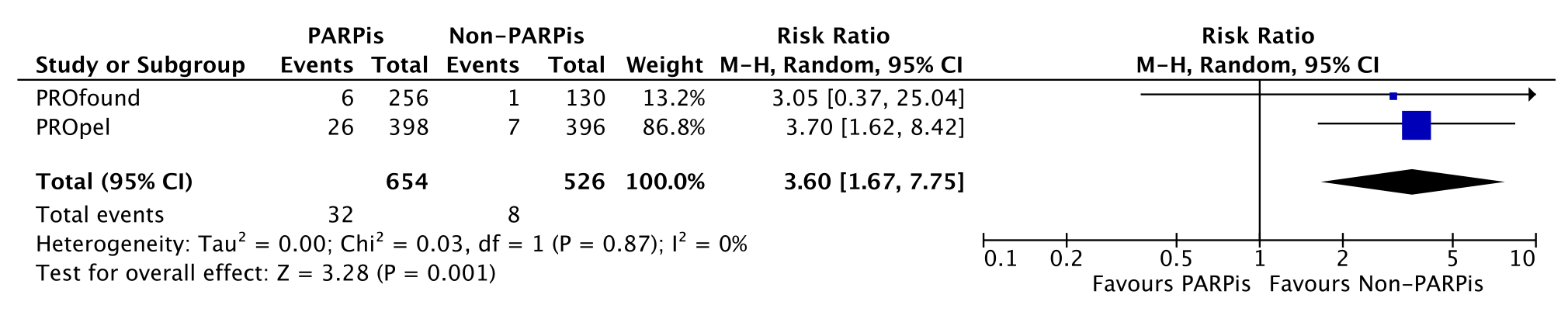 |

**Supplementary Figure 2. Relative risk of: all grades myocardial infarction (A), high-grade myocardiac infarction (B), all grades cardiac failure (C), high-grade cardiac failure (D), all grades venous thromboembolism (E), high-grade venous thromboembolism (F), all grades pulmonary embolism (G), high-grade pulmonary embolism (H) of PARPis versus Non-PARPis in mCRPC.**

CI: confidence interval

A

| 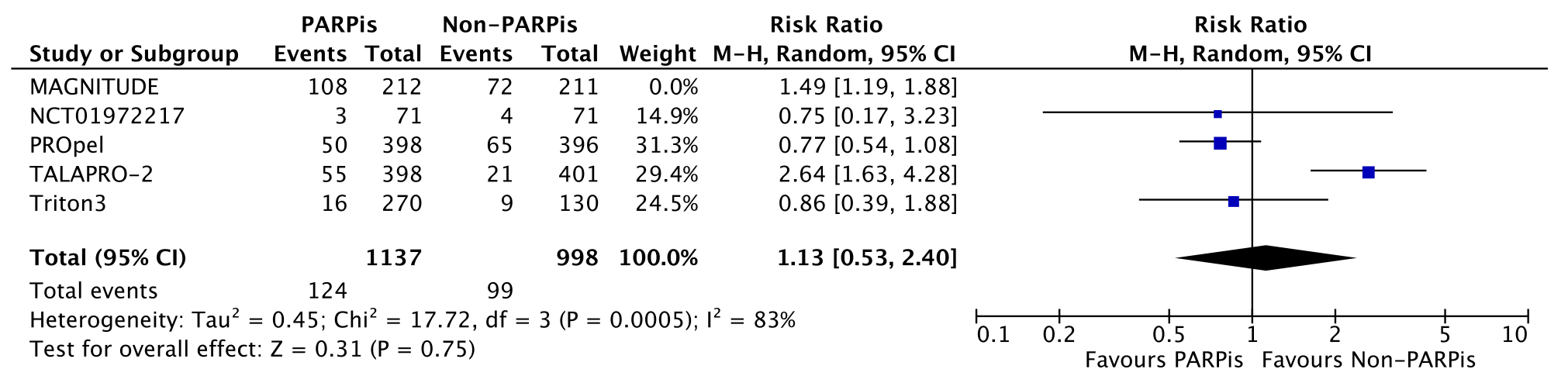 | 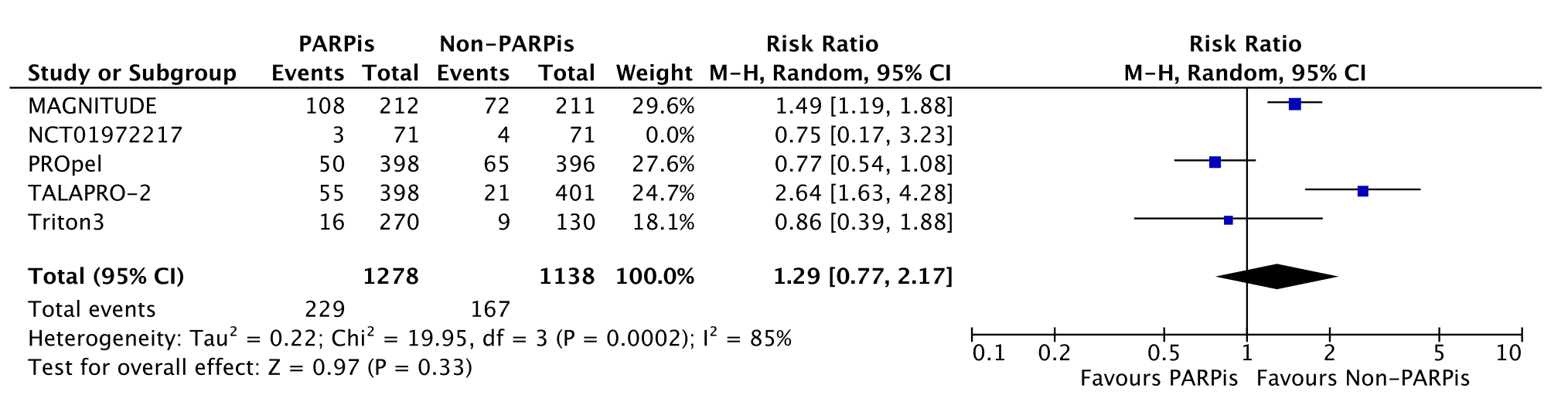 |
| --- | --- |
| 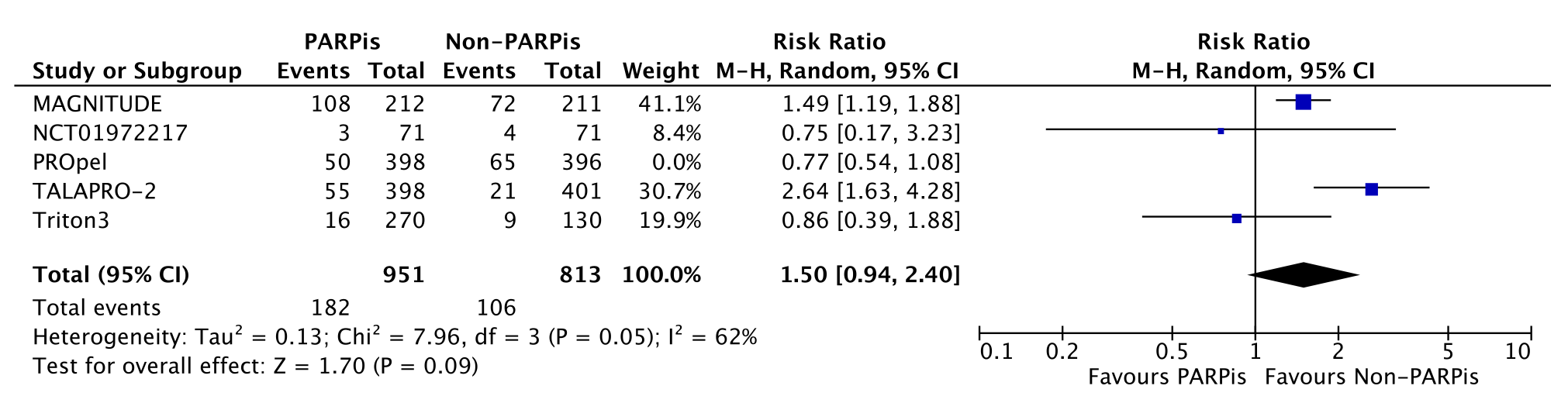 | 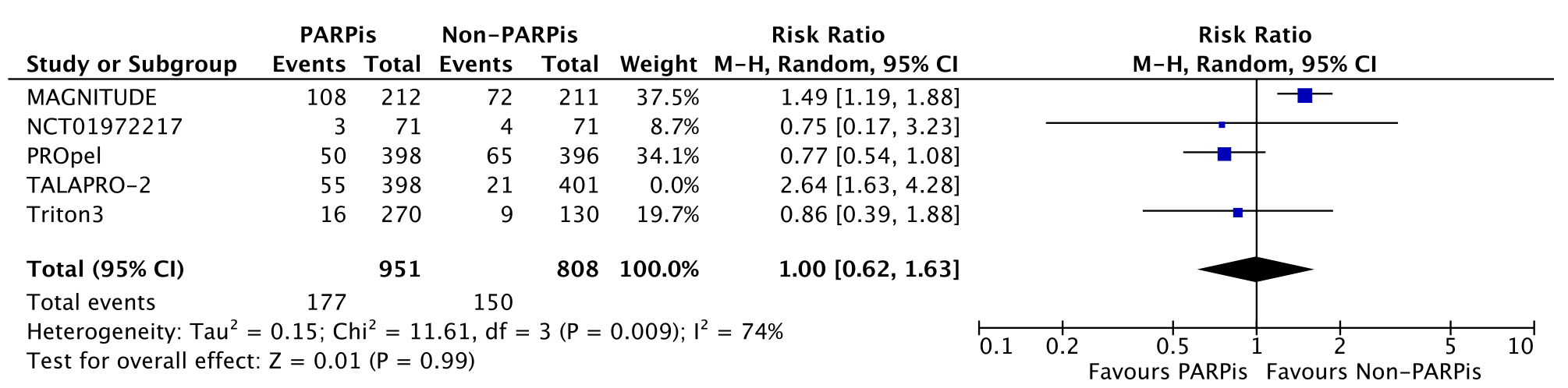 |
| 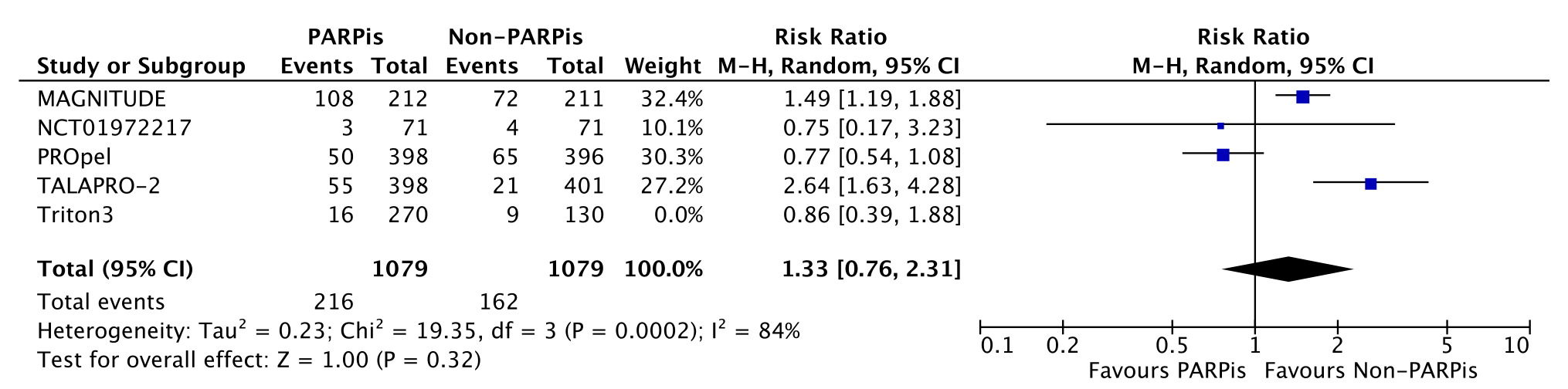 |  |

B

| 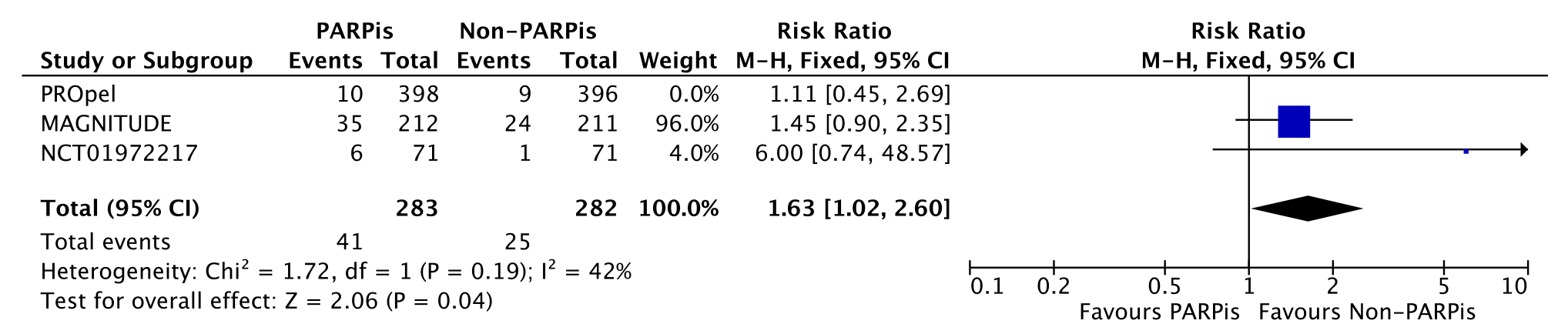 | 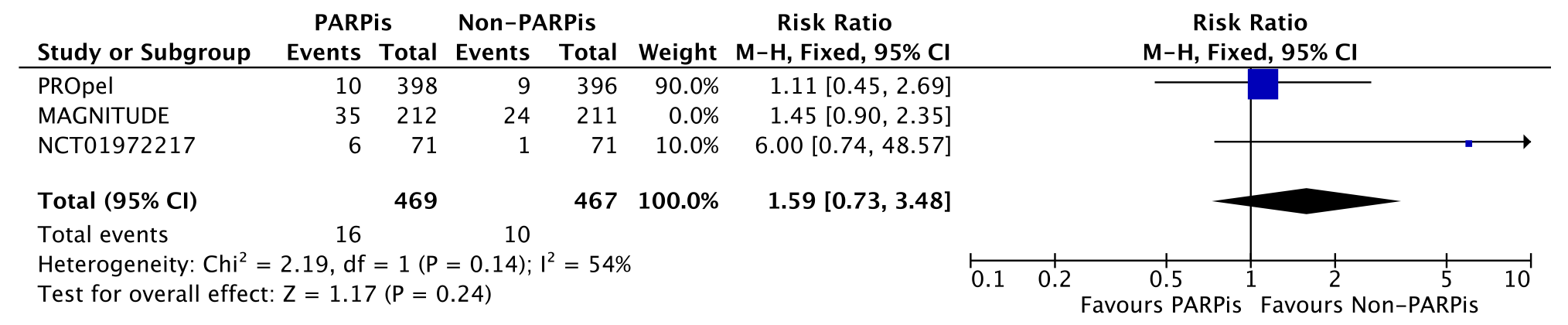 |
| --- | --- |
| 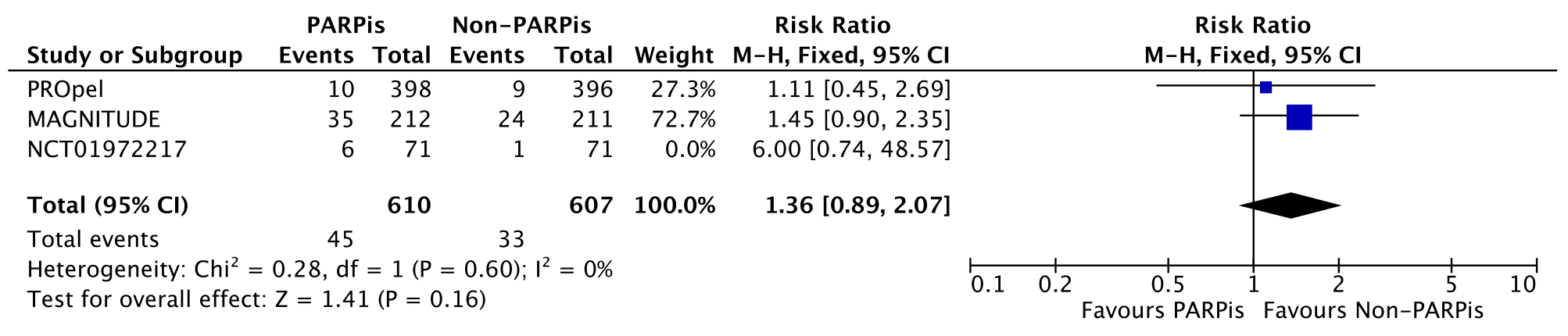 |  |

C

| 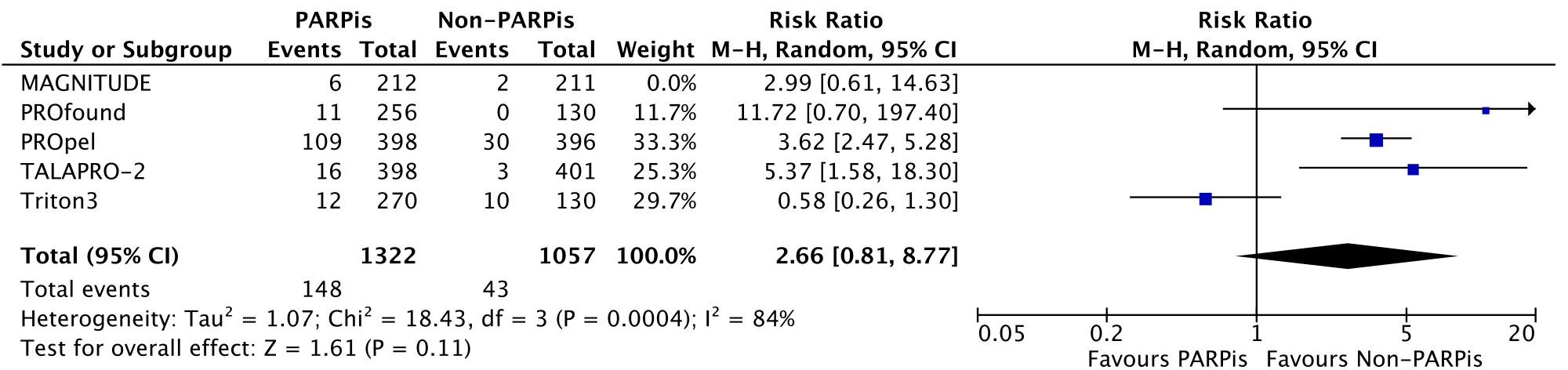 | 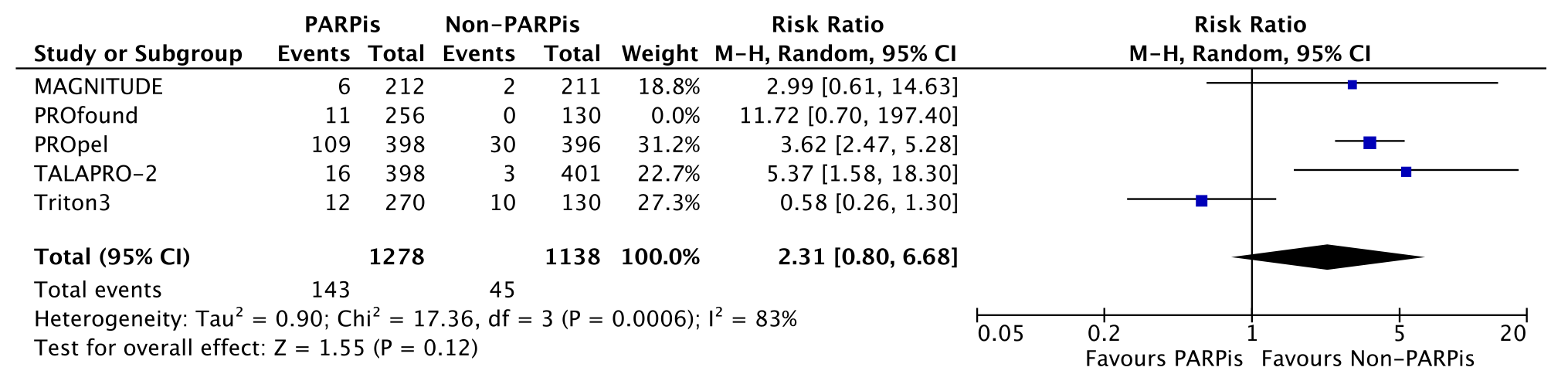 |
| --- | --- |
| 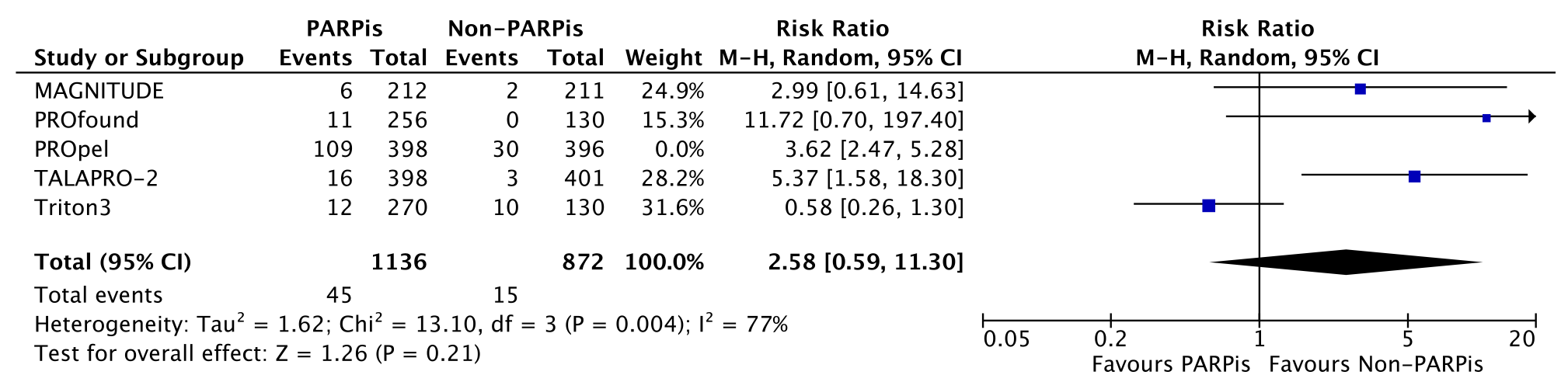 | 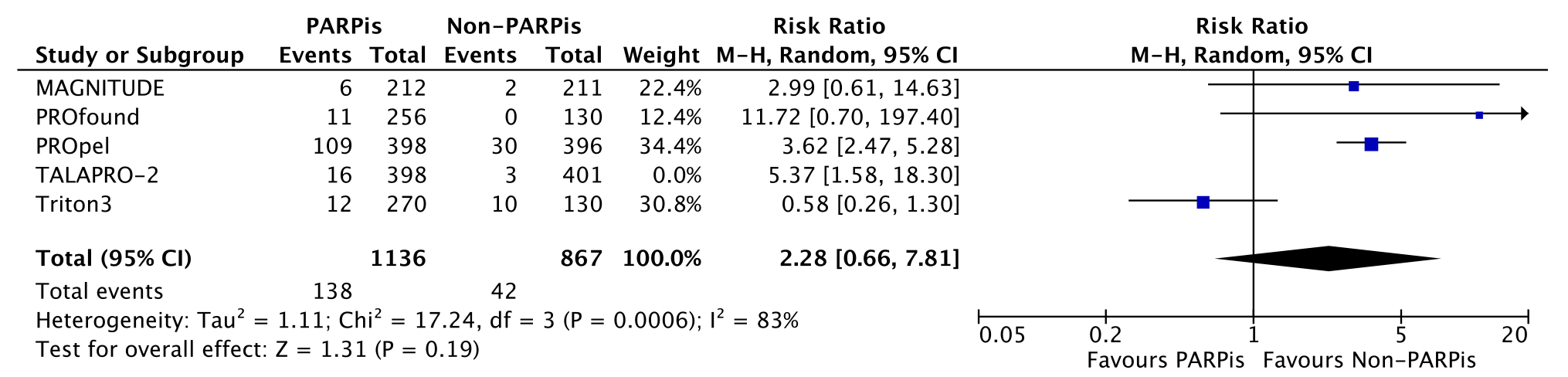 |
| 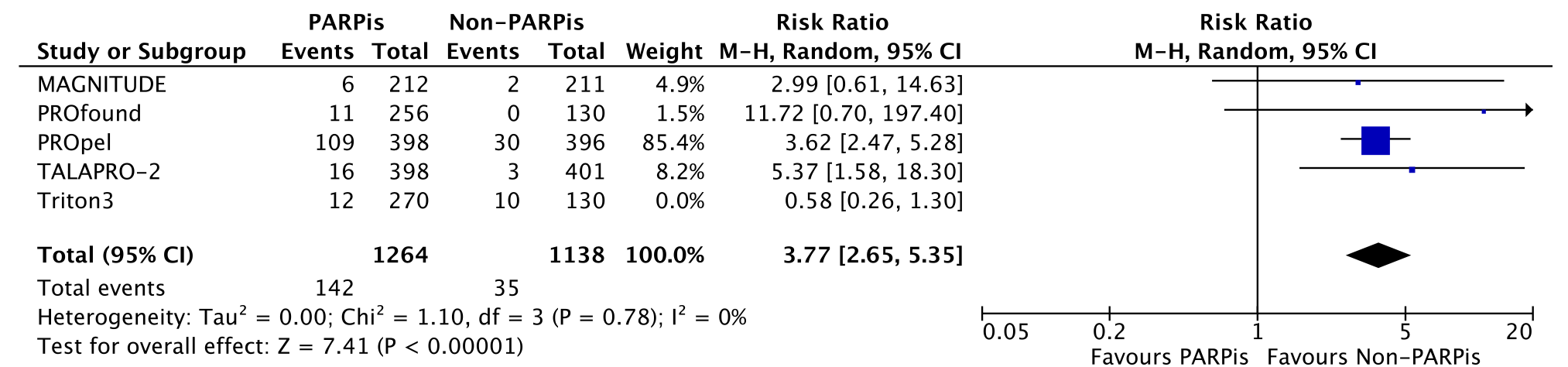 |  |

D

| 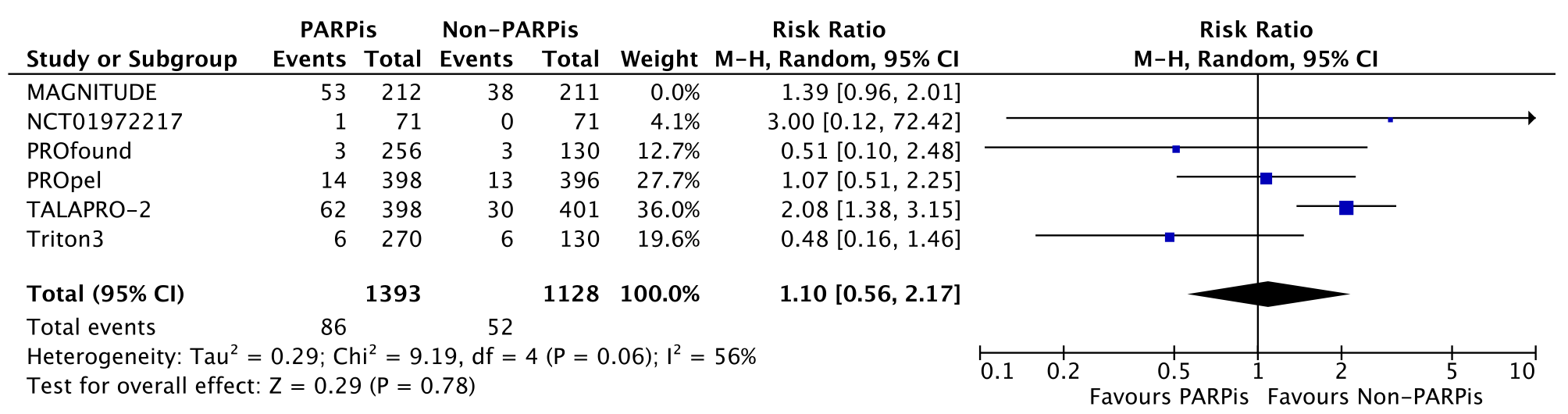 | 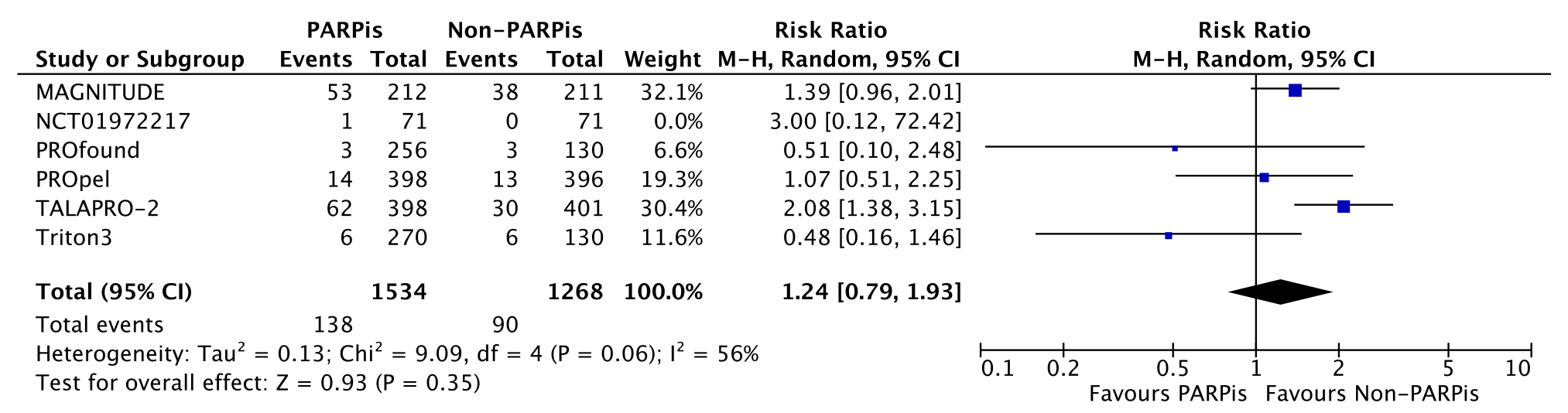 |
| --- | --- |
| 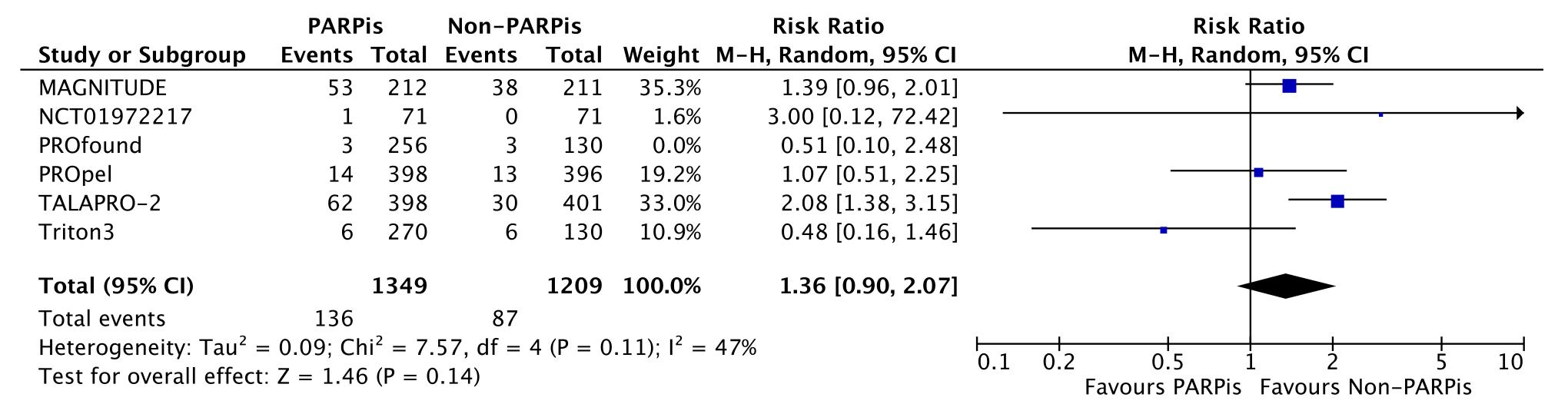 | 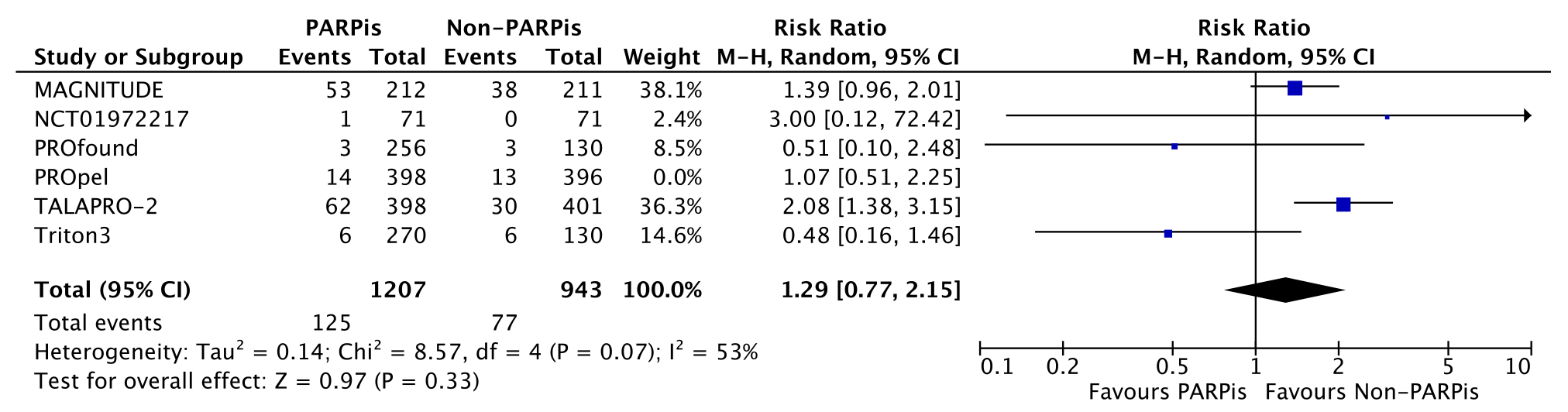 |
| 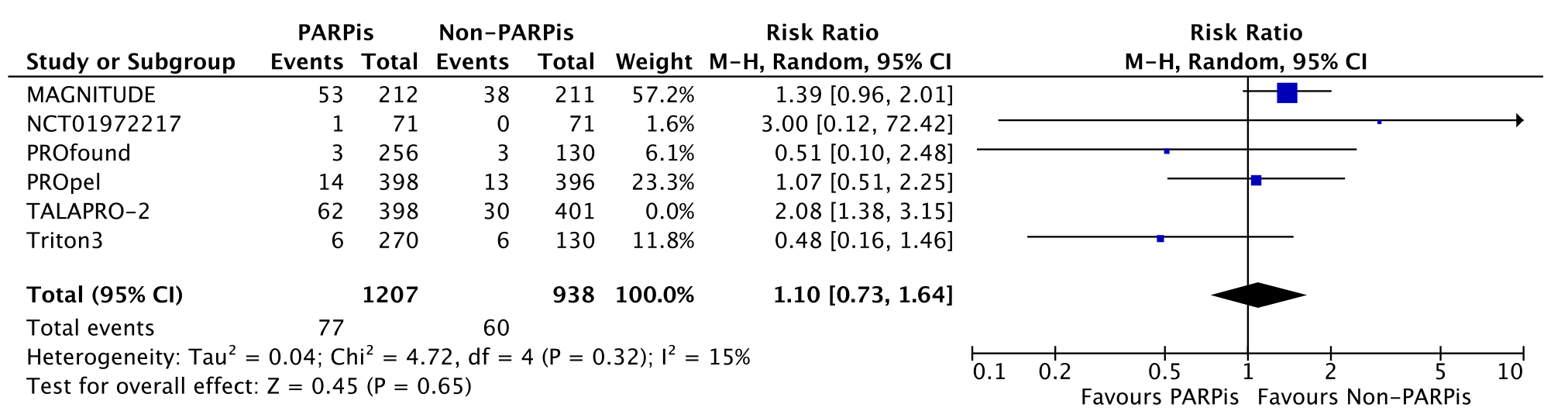 | 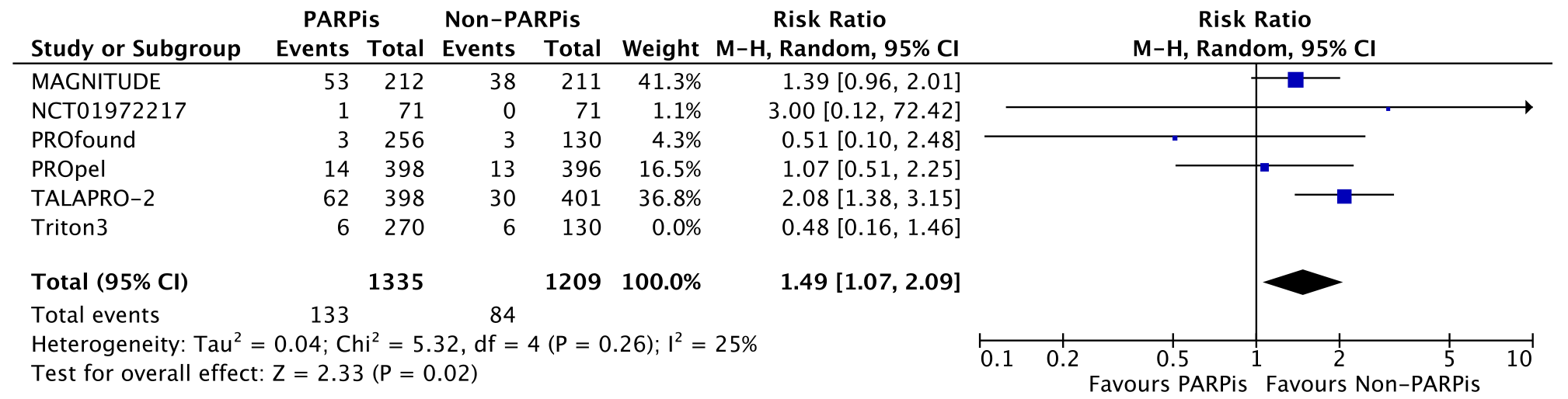 |

E

| 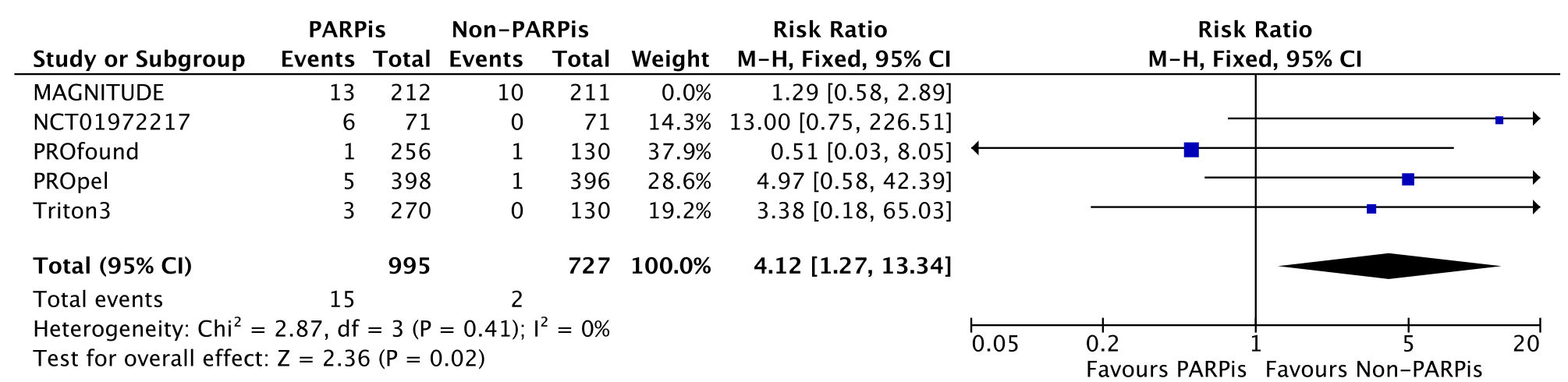 | 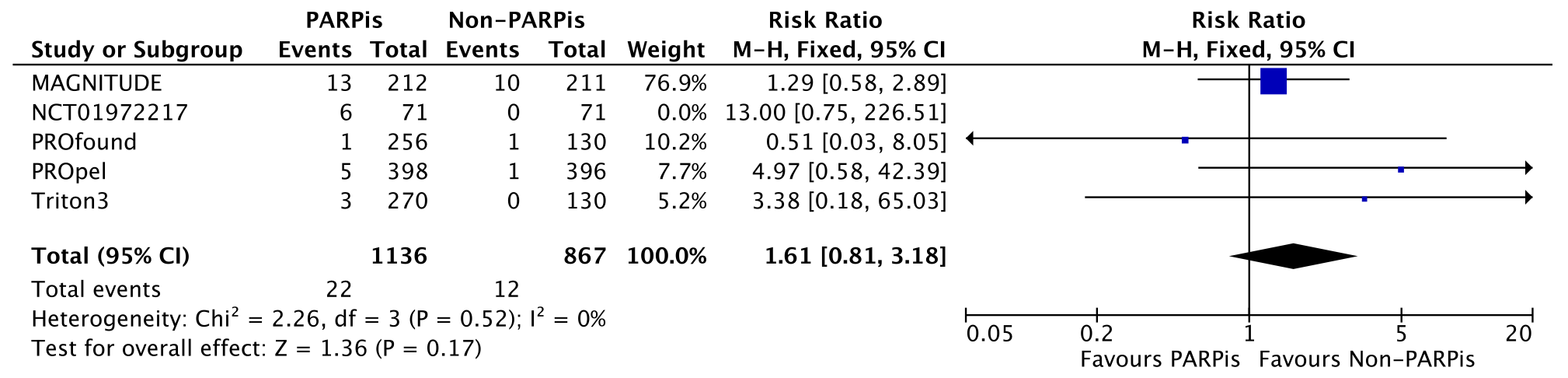 |
| --- | --- |
| 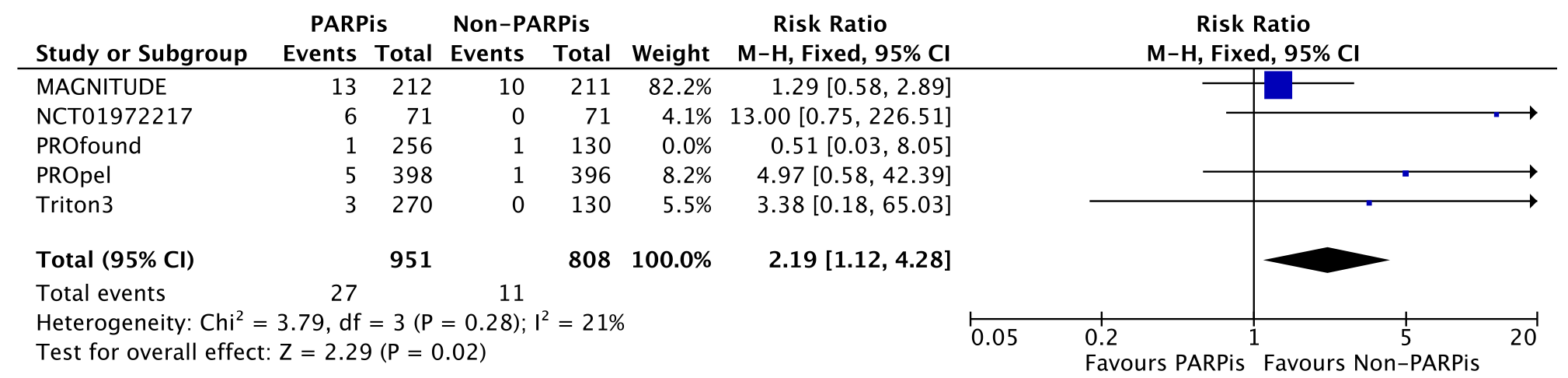 | 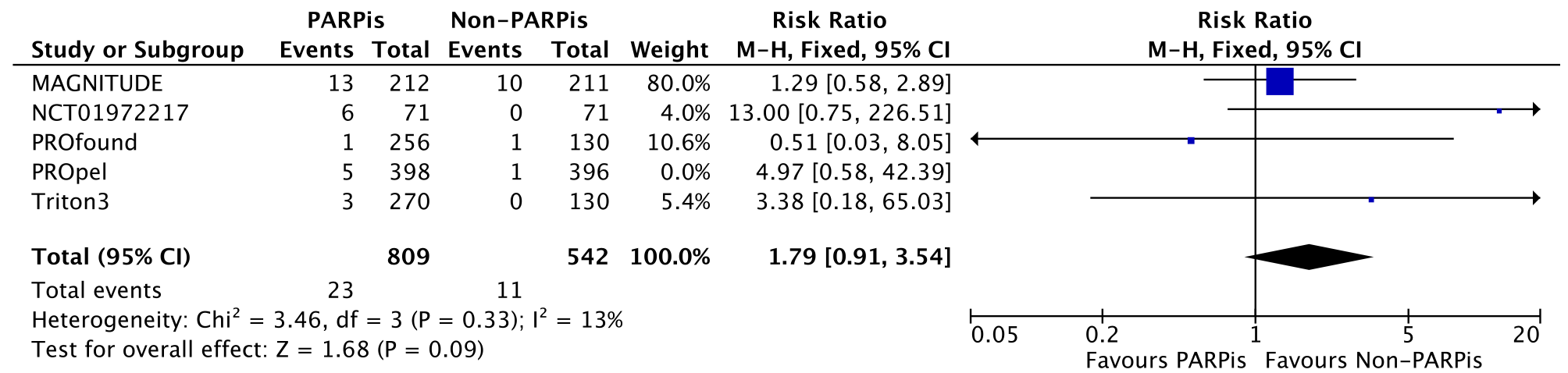 |
| 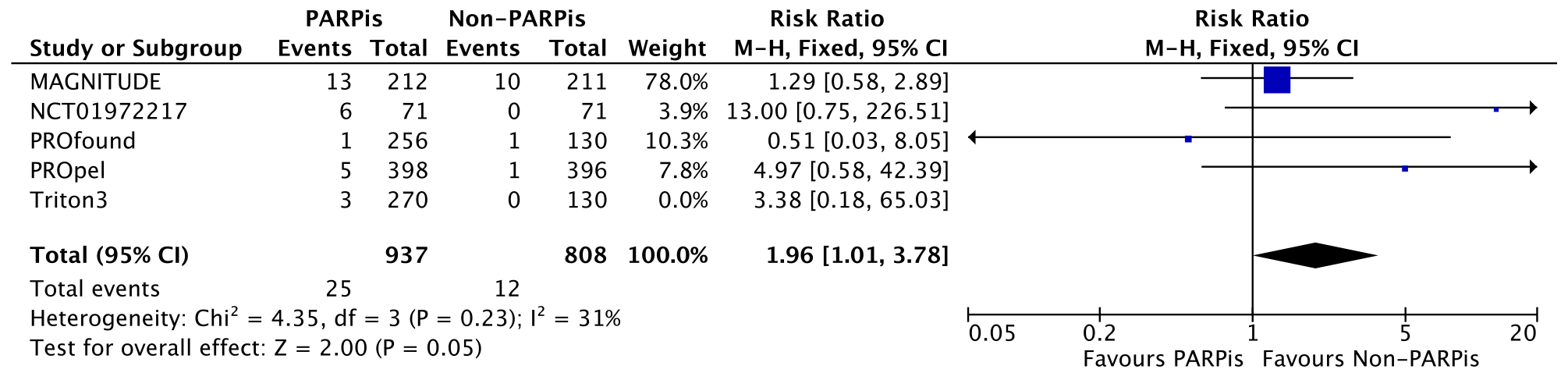 |  |

F

| 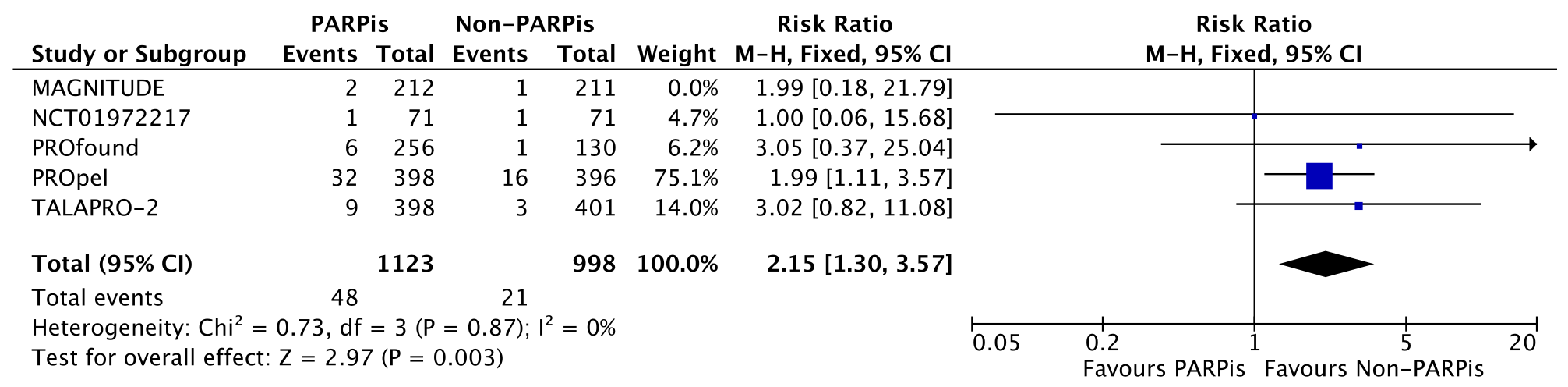 | 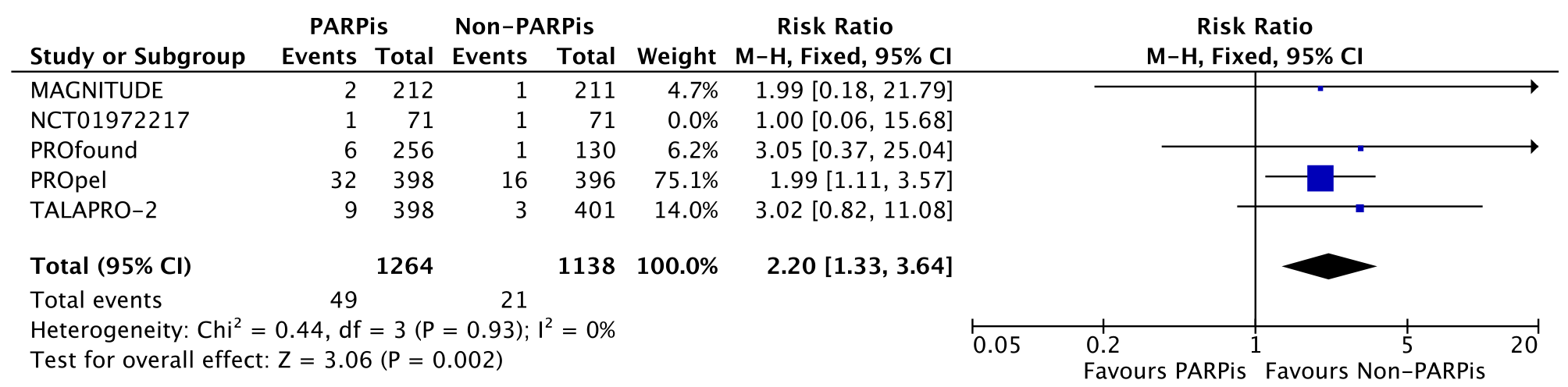 |
| --- | --- |
| 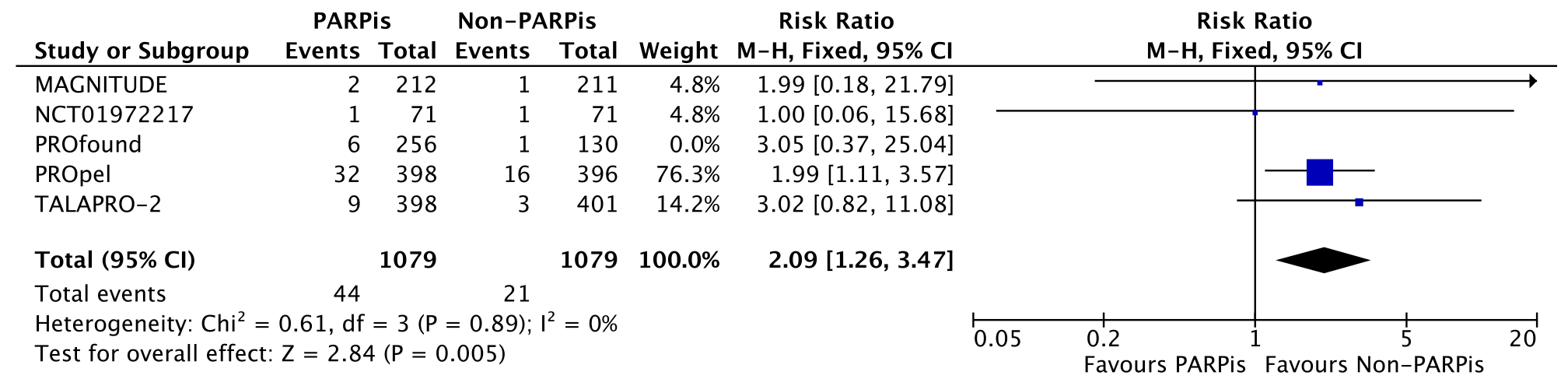 | 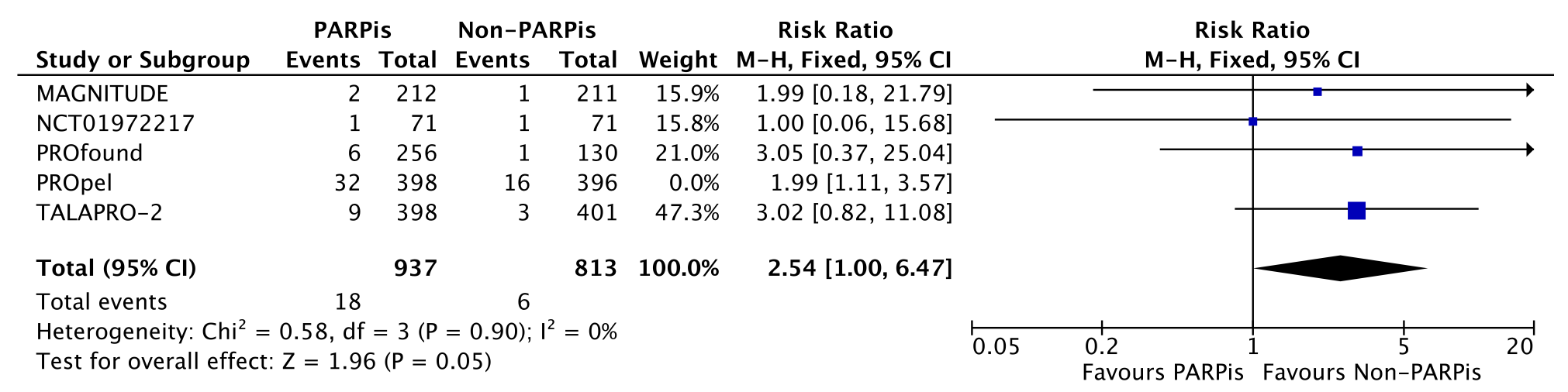 |
| 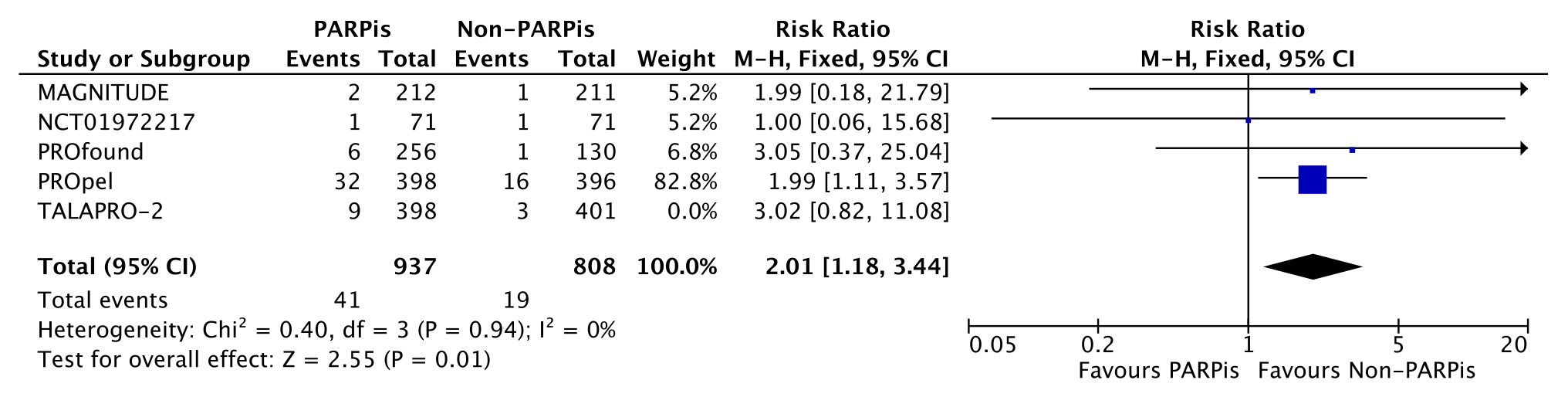 |  |

**Supplementary Figure 3. Sensitivity analysis of: all grades hypertension (A), all grades MACEs (B), all grades thromboembolic AEs (C), high-grade hypertension (D), high-grade MACEs (E), high-grade thromboembolic AEs (F) of PARPis versus Non-PARPis in mCRPC.**

CI: confidence interval
